# pH-Dependent Partitioning of Ionizable Organic Chemicals between the Silicone Polymer Polydimethylsiloxane (PDMS) and Water

**DOI:** 10.1021/acsenvironau.1c00056

**Published:** 2022-02-16

**Authors:** Lili Niu, Luise Henneberger, Julia Huchthausen, Martin Krauss, Audrey Ogefere, Beate I. Escher

**Affiliations:** † Department of Cell Toxicology, UFZ − Helmholtz Centre for Environmental Research, 04318 Leipzig, Germany; ‡ Key Laboratory of Pollution Exposure and Health Intervention of Zhejiang Province, Interdisciplinary Research Academy (IRA), Zhejiang Shuren University, Hangzhou 310015, China; § Department of Effect Directed Analysis, Helmholtz Centre for Environmental Research, 04318 Leipzig, Germany; ∥ Center for Applied Geoscience, Eberhard Karls University of Tübingen, Schnarrenbergstr. 94-96, 72076 Tübingen, Germany

**Keywords:** passive equilibrium sampling, acidity constant, partition constant, speciation, microplastic

## Abstract

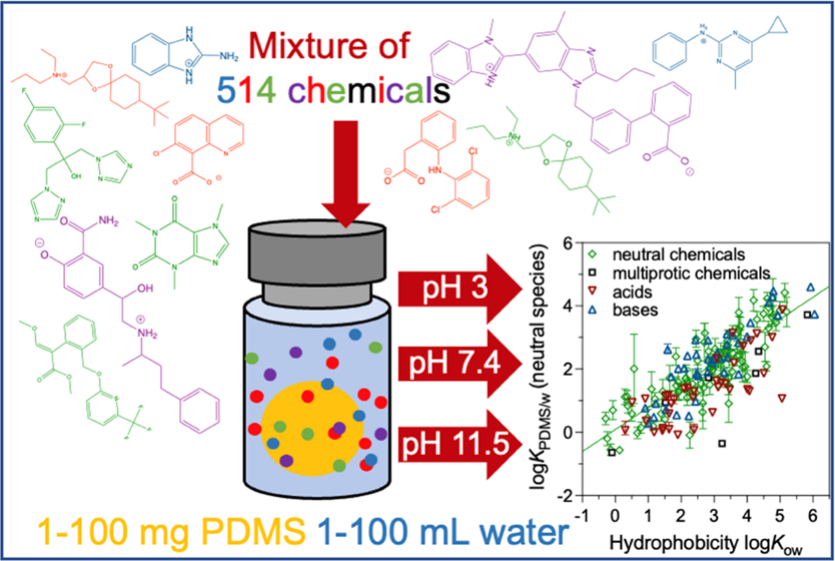

The silicone polymer polydimethysiloxane (PDMS) is a popular passive sampler for *in situ* and *ex situ* sampling of hydrophobic organic chemicals. Despite its limited sorptive capacity for polar and ionizable organic chemicals (IOC), IOCs have been found in PDMS when extracting sediment and suspended particulate matter. The pH-dependent partitioning of 190 organics and IOCs covering a range of octanol–water partition constants log *K*_ow_ from −0.3 to 7.7 was evaluated with a 10-day shaking method using mixtures composed of all chemicals at varying ratios of mass of PDMS to volume of water. This method reproduced the PDMS–water partition constant *K*_PDMS/w_ of neutral chemicals from the literature and extended the dataset by 93 neutral chemicals. The existing quantitative structure–activity relationship between the log *K*_ow_ and *K*_PDMS/w_ could be extended with the measured *K*_PDMS/w_ linearly to a log *K*_ow_ of −0.3. Fully charged organics were not taken up into PDMS. Thirty-eight monoprotic organic acids and 42 bases showed negligible uptake of the charged species, and the pH dependence of the apparent *D*_PDMS/w_(pH) could be explained by the fraction of neutral species multiplied by the *K*_PDMS/w_ of the neutral species of these IOCs. Seventeen multiprotic chemicals with up to three acidity constants p*K*_a_ also showed a pH dependence of *D*_PDMS/w_(pH) with the tendency that the neutral and zwitterionic forms showed the highest *D*_PDMS/w_(pH). *D*_PDMS/w_(pH) of charged species of more hydrophobic multiprotic chemicals such as tetrabromobisphenol A and telmisartan was smaller but not negligible. Since these chemicals show high bioactivity, their contribution to mixture effects has to be considered when testing passive sampling extracts with *in vitro* bioassays. This work has further implications for understanding the role of microplastic as a vector for organic micropollutants.

## 1 Introduction

Passive equilibrium sampling (PES) has been identified as a robust and promising method for monitoring the freely dissolved concentrations of chemicals in water and the bioavailable fractions in particles. Among diverse passive samplers, silicone (polydimethylsiloxane, PDMS) is a popular absorbent phase that has been widely used in PES devices for in situ passive sampling in water^1,2^ and sediment,^3^ as well as for ex situ equilibrium partitioning in the laboratory.^4^ To quantify the freely dissolved concentrations of contaminants of interest with PES techniques, the PDMS–water partition constant (*K*_PDMS/w_) is a critically important parameter. The partitioning of contaminants depends on the material of the passive samplers, the conditions of sampling/equilibration, as well as the physicochemical properties of target analytes.^5^ PDMS has been recommended as a passive sampler for hydrophobic and nonionized chemicals because of its limited enrichment of hydrophilic chemicals with log *K*_ow_ (octanol–water partition constant) lower than 3.^6^

As both the log *K*_PDMS/w_ and the logarithm of the sediment–water partition constant log* K*_sed/w_ are linearly correlated with the log *K*_ow_, the logarithms of the PDMS–sediment partition constants log *K*_PDMS/sed_ are fairly independent of the log *K*_ow_.^7^ This feature has been exploited to apply PDMS extracts from sediments in *in vitro* bioassays because the mixture effect equivalents in PDMS can be directly translated into effect equivalents in sediments without the need to know the composition of the mixture.^8−11^ This method has also been applied for biota, such as fish tissue,^12−15^ dugong blubber,^16^ turtle blood,^17^ and human tissue and blood^18^ because the PDMS–lipid partition constants *K*_PDMS/lipid_ are also virtually independent of hydrophobicity.

This relationship might not be as straightforward for ionizable organic compounds (IOCs), which have two or more species, and one or multiple charges. IOCs are an important group of environmental organic pollutants with a great concern related to bioaccumulation^19^ and ecotoxicity.^20^ In our recent study on the bioavailable fractions of chemicals in sediments, it was surprising to find that some IOCs like benzethonium, bis(2-ethylhexyl) phosphate, dinoseb, naproxen, 2,4-dichlorobenzoic acid, perfluorohexanoic acid, 2-benzothiazole sulfonic acid, and 2-naphthalene sulfonic acid were detected in PDMS extracts,^11^ albeit at rather low concentrations. Ahrens et al.^2^ observed that 86 pesticides covering the log *K*_ow_ of 0.7–7.0 including IOCs were capable to accumulate in silicone rubber sheets.

This topic is also of relevance for (micro)plastic research. Plastic may act as a vector for micropollutants.^21−24^ Sorption of charged molecules such as perfluorooctanesulfonate to polyethylene (PE), polystyrene (PS), and polyvinylchloride (PVC) had been reported,^25^ while other studies found that the role of the charged species for partitioning to PE and PS was negligible^26^ or relatively low for the carboxylic acids naproxen, ibuprofen, and diclofenac.^27^ Other studies explained the low affinity of the ionic species of IOCs to PE, polypropylene (PP), and PVC as an adsorptive process.^28^

The conventional wisdom is that the partitioning of IOCs into polymers is correlated with their speciation and only the neutral species is expected to partition into PDMS. The interaction of different species in complex mixtures might result in the formation of ion pairs and the partitioning of net-neutral ion pairs and complexes might be a reason for charged organics to partition into PDMS.

To evaluate the distribution of IOCs between PDMS and water in complex mixtures, a total of 514 chemicals that have been previously found in water^29^ or sediments^11^ and that cover a broad range of physicochemical properties with hydrophobicity log *K*_ow_ ranging from −3.6 to 9.7 and speciation were spiked to water to determine their partitioning to PDMS at different pH (pH 3.0, 7.4, and 11.5). Our objectives were (1) to validate this simple mixture method for the quantification of *D*_PDMS/w_ at different pH values, (2) to explore the influence of pH on the speciation of IOCs and the subsequent partitioning into PDMS, (3) to evaluate if charged organics can be taken up into PDMS if present in complex mixtures, and (4) to derive the *K*_PDMS/w_ for neutral chemicals and the neutral species of IOCs via the apparent distribution ratios between PDMS and water (*D*_PDMS/w_). We further discuss the results in light of microplastic research and the use of PES in combination with bioassays.

## 2 Materials and Methods

### 2.1 Chemicals

The standard mixture of 514 organic micropollutants was prepared from individual methanolic stock solutions at 1 μg/mL. It had served previously for chemical analysis in water^29^ and sediments^11^ and covered a wide range of physicochemical properties. Names, identifiers, and physicochemical properties of these chemicals are listed in Table S1. All solvents were of UPLC grade (Honeywell). ACS grade of phosphoric acid (85–90%), sodium dihydrogen phosphate monohydrate (Merck, Darmstadt, Germany), disodium hydrogen phosphate dihydrate (Merck, Darmstadt, Germany), and trisodium phosphate dodecahydrate (Chemsolute, Th. Geyer GmbH & Co. KG, Renningen, Germany) were used to prepare phosphate buffer. Nile blue A (Sigma-Aldrich), phenol red (Sigma-Aldrich), and bromophenol blue (Alfa Aesar) were used as indicators for pH measurement. Other reagents used for pH measurement were of analytical grade.

### 2.2 Phosphate Buffer

Stock solutions of phosphoric acid, sodium dihydrogen phosphate monohydrate, disodium hydrogen phosphate dihydrate, and trisodium phosphate dodecahydrate were prepared individually with Milli-Q water to 10 mM. The stock solutions were mixed until achieving 10 mM phosphate buffer at pH of 3.0, 7.4, and 11.5. The pH in the buffer was measured with a Prolab 2500 pH meter from SI Analytics equipped with an A162 pH electrode and an A157 pH microelectrode before adding the chemical mixture stocks.

### 2.3 pH Measurements

The pH of the water phase in the presence of chemicals before and after equilibration with PDMS was determined using an absorbance method on 96-well plates with a Tecan plate reader. The pH 3.0 experiments were monitored with bromophenol blue, the pH 7.4 experiments with phenol red, and the pH 11.5 experiments with Nile blue A. Concentrated bromophenol blue was prepared in ethanol at 3 mg/mL, phenol red was prepared in Milli-Q water at a concentration of 3 mg/mL, and Nile blue A in ethanol at 2 mg/mL. Ethanol was used to increase the solubility of bromophenol blue and Nile blue A in water samples. Prior to measurements, 1 μL of concentrated dyes was added to 96-well plates containing 200 μL of corresponding water samples. The wavelengths used for measurement were optimized with a full scanning range from 350 to 700 nm or from 420 to 570 nm using calibration samples (Figure S1). Phenol red had maximum absorbance at 430 and 560 nm, bromophenol blue at 435 and 590 nm, and Nile blue A at 520 and 645 nm. The ratios of the maximum absorbance were used for plotting calibration regressions (Figure S1).

### 2.4 Determination of Acidity Constants

Acidity constants (p*K*_a_) of selected chemicals were measured with a Sirius T3 automated titrator (Pion) equipped with a glass Ag/AgCl pH electrode, a UV dip probe, a temperature probe, and a stirring unit. All experiments were performed at 25 °C with a constant ionic strength of 0.15 M KCl under an argon atmosphere using either a spectrophotometric method or a potentiometric method for chemicals without a chromophore close to the ionization center.

The spectrophotometric method measured the pH-dependent change in the UV absorption spectra of chromophores located in close proximity to ionizable groups to determine the proportion of different UV-active species as a function of pH and thereby to derive the p*K*_a_.^30−32^ Five microliters of a 10 mM DMSO stock solution of each test chemical were transferred to a Sirius T3 test vial. Twenty-five microliters of mid-range phosphate buffer (14.4 mM K_2_HPO_4_ and 0.15 M KCl) were added to each vial. A reference vial with 5 μL of DMSO (Roth, A994–100 ML) and 25 μL of mid-range buffer was prepared for each sample vial. The p*K*_a_ measurement was performed using the automated UV-metric p*K*_a_ protocol of Sirius T3 Control software (Version 2.0.0.0.). Three sequential titrations were performed with the same sample in the range of pH 1.5 to 12.5 by adding 0.5 M HCl and 0.5 M KOH while UV absorption was measured. The titration direction was chosen based on the chemical class so that the test chemical had the highest solubility at the beginning (low-to-high pH for bases and high-to-low pH for acids).

The potentiometric method measured the p*K*_a_ from the ionization change of the sample (0.5–2 mg) at different pH values^33−35^ using the automated pH-metric p*K*_a_ protocol of Sirius T3 Control software. The titration range was from pH 2 to 12 for the potentiometric method, and all other conditions were the same as described above.

Data evaluation was performed using Sirius T3 Refine software (version 2.0.0.0.). All p*K*_a_ values were reported as a mean value of the three replicate titrations.

For chemicals with poor water solubility, the titration was performed in the presence of 20–50% ionic strength-adjusted methanol under the same conditions as described above. The automated UV-metric *p*_s_*K*_a_ or the pH-metric *p*_s_*K*_a_ protocol was used for the titration. Data evaluation was performed using Sirius T3 Refine software (version 2.0.0.0.). The apparent p*K*_a_ measured in the presence of methanol was extrapolated to the p*K*_a_ at 0% methanol using the Yasuda–Shedlovsky extrapolation.^31,35−38^ Titrations at a minimum of three different methanol–water ratios were necessary for a reliable extrapolation.

### 2.5 Partitioning Experiments

There were 65 different experiments with a range of conditions to capture not only a diverse range of speciation but also a range of PDMS mass to water volume ratios: three different pH values, three different water volumes, three different PDMS masses, four different spiked concentrations, and various blanks. Originally, there were 66 experiments but the sample at pH 3, 10 days, 1 mg of PDMS and 10 mg/mL spiked was an outlier and excluded from data evaluation. The PDMS sheets of 0.125 mm thickness were cleaned with ethyl acetate using Soxhlet extraction devices for 16 hours before use. After drying and weighing, the PDMS sheets were placed in amber vials with phosphate buffer of different pH. To cover chemicals with different *K*_ow_ in different scenarios, PDMS samples of approximately 1 mg (0.86 μL), 10 mg (8.6 μL) and 100 mg (86 μL) were prepared. The ratios of PDMS to water (kg/L) were theoretically set as 1:10; 1:100; 1:1000, 1:10 000, and 1:100 000. The masses of PDMS sheets used in each vial were ranging from approximately 1 to 100 mg with the exact masses in each vial given in Table S2, and the phosphate buffer volumes ranged from 1 to 100 mL. The concentrations of chemical mixtures spiked to water were 5, 10, and 50 ng/mL in the samples with PDMS-to-water ratios 1:10; 1:100, and 1:1000, while only 5 ng/mL was spiked in the 1:10 000 samples and 1 ng/mL in the 1:100 000 samples. Blanks with PDMS-to-water ratios of 1:10 and 1:100 at three pH levels were prepared in parallel. Additionally, 200 μL solutions with different levels of spiked chemicals were also prepared for pH determination to check if the pH of the solutions was changed by the presence of spiked chemical mixtures. After crimping with aluminum caps, the vials were shaken at 250 rpm using an orbital shaker at room temperature in the dark. To check that the equilibrium between water and PDMS sheets was attained, the vials were incubated for 5 and 10 days.

At the set time points, the solutions in each vial were taken out and divided into two aliquots. One aliquot was used for pH measurement and the other aliquot was acidified to pH around 2 with concentrated phosphoric acid prior to instrumental analysis. The PDMS sheets were washed, dried, and extracted according to the previously established method,^11^ and the PDMS extracts were redissolved in 250 μL of methanol.

### 2.6 Instrumental Analysis

A liquid chromatography (LC) instrument equipped with a Thermo Ultimate 3000 liquid chromatography system (Thermo) and a heated electrospray ion source to a quadrupole orbitrap mass spectrometer (MS, Thermo QExactive Plus) was used for the quantification of chemicals in PDMS extracts and in water. Detailed instrumental conditions including the column information and gradient elution program can be found in Niu et al.^11^ The positive and negative modes of ionization were recorded during the same run. Prior to analysis, the mixture of 37 internal standards as used in our previous study^11^ was spiked in each sample. The calibration samples were prepared in both phosphate buffer of pH 2 and methanol to match with the analyzed samples. To check the carry-over and instrument performance, solvent blanks, procedure blanks, and quality control samples were run with every batch of samples. TraceFinder 5.1 General Quan (Thermo) was deployed to quantify the chemical concentrations in samples based on an internal standard calibration method.

### 2.7 Data Evaluation

The 475 chemicals with acceptable calibration curves (*R*^2^ > 0.96) in chemical analysis were further scrutinized according to the flow chart depicted in Figure S2. The concentration of chemical *i* in extracts was converted to the amount in PDMS *n*_PDMS,*i*_ (g) and in water *n*_w,*i*_ (g). The blanks were subtracted from the level of the *n*_PDMS,*i*_ and *n*_w,*i*_. If the blanks *n*_PDMS,blank_ or *n*_w,blank_ were more than 50% of the chemical signal, the chemical was excluded. Then, the mass balance *n*_tot,*i*_ = *n*_PDMS,*i*_ + *n*_w,*i*_ was checked. Only chemicals that were detected in both phases with a mass balance of *n*_tot,*i*_ = 100 ± 50% × *n*_spiked,*i*_ were included in the further data evaluation (Figure S2). Only data with 0.01 < *n*_PDMS,*i*_/*n*_w,*i*_ < 100 were included for derivation of *D*_PDMS/w,i_ because if the difference between the *n*_PDMS,*i*_ and *n*_w,*i*_ was more than two orders of magnitude, the robustness of the resulting *D*_PDMS/w,*i*_ would be low. If partition experiments are planned for single chemicals, one typically chooses even a narrower range of one order of magnitude.

The concentrations of chemical *i* in PDMS (*C*_PDMS,*i*_) and in water (*C*_w,*i*_) were calculated by dividing the masses of chemicals in PDMS (*n*_PDMS,*i*_) and in water (*n*_w,*i*_) by the mass of PDMS (*m*_PDMS_) and volume of water (*V*_w_), respectively.
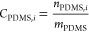
1
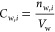
2The apparent *D*_PDMS/w,*i*_ for chemical *i* can be derived from the slope of the linear regression with paired chemical concentrations detected in PDMS (*C*_PDMS,*i*_, ng/g) and the water phase (*C*_w,*i*_, ng/mL) or as their concentration ratio (eq 3).
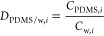
3For neutral chemicals, the *D*_PDMS/w,*i*_ should be independent of pH, as will be checked below. The log *K*_PDMS/w,*i*_ for a neutral chemical *i* was calculated as the average of log *D*_PDMS/w,i_ of all measurements.

For IOCs with one ionizable function, the apparent *D*_PDMS/w,*i*_ was determined by the fractions of the neutral (α_neutral,*i*_) and charged (1 – α_neutral,*i*_) species (eq 4).
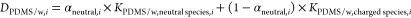
4
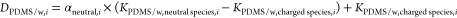
5If the *D*_PDMS/w,*i*_ is plotted against α_neutral,*i*_, the *K*_PDMS/w,charged species,*i*_ and *K*_PDMS/w,neutral species,*i*_ can be derived from the slope and *y* intercept of the linear regression (eq 5). If *K*_PDMS/w,charged species,*i*_ is close to 0, that means no charged species partition into PDMS and the *D*_PDMS/w,*i*_ is proportional to the fraction of neutral species at a given pH level. If the *K*_PDMS/w,charged species,*i*_ > 0, this could be caused by the formation of ion pairs, where the charged species enter into PDMS as net-neutral ion pairs or adsorb to the surface.

The fraction of species *j* (α*_j_*) is controlled by the acid dissociation constant (p*K*_a_) and the pH. The fractions of the two species (α_1_ and α_2_) of monoprotic acids and bases can be calculated with eqs 6–7. For acids, α_1_ is the fraction of neutral species α_neutral,*i*_, while α_2_ represents the α_neutral,*i*_ of bases.
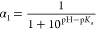
6
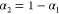
7The fraction of three species (α_1_, α_2_, and α_3_) of diprotic acids and bases can be calculated by eqs 8–10 at a given pH level. For chemicals with one basic p*K*_a_ and one acidic p*K*_a_, α_2_ is the fraction of neutral or zwitterionic species α_neutral,*i*_ and α_3_ is the fraction of neutral or zwitterionic species for those with two basic p*K*_a_. General equations for multiprotic IOCs with more than two p*K*_a_ values can be found in the Appendix of Escher et al.^20^
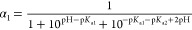
8
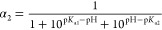
9
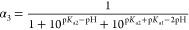
10

## 3 Results and Discussion

### 3.1 Stability of pH in Solutions After Spiking Chemical Mixtures and After Passive Equilibrium Sampling

The stability of pH throughout the whole passive sampling is a crucial factor that influences the reliability of experiments with IOCs. After spiking with chemical mixtures and before PES, the pH of the phosphate buffers ranged from 3.1 to 3.3, from 7.2 to 7.3, and from 10.8 to 11.6 for the targeted pH values of 3, 7.4, and 11.5, respectively (Figure S3). The influence of spiked chemical mixtures on the solution pH was limited, with the general variation less than 10% at day 0. Except for pH 11.5, where the pH dropped by one unit upon addition of 1 ng/mL for each chemical in the chemical mixture, the pH values of spiked solutions were similar to the pH in the blanks (pH 3.2 and 7.2 in blanks). No significant pH drift occurred for samples during partitioning with the pH of 3.3 ± 0.10 (3.3 ± 0.10 in blank), 7.3 ± 0.05 (7.3 ± 0.06 in blank), and 10.4 ± 0.26 (10.3 ± 0.05 in blank) after 5 and 10 days of equilibration. The pH of the water phase during the equilibration was much more stable in the group of pH 7.4 (Figure S3B) than the other two pH values (Figure S3A,C). Generally, the phosphate buffer maintained the pH of the water phase even after spiking with hundreds of chemicals and after 10 days of incubation.

### 3.2 Mass Balances and Losses due to Hydrolysis or Other Processes

Four hundred seventy-five chemicals were quantified (Table S2) but 199 chemicals were not detected in PDMS at all or in very low amounts in a few samples (Table S3). These were mainly fully charged chemicals at the given pH. The alkylammonium cations were often detected in the blanks and the concentrations in PDMS did not differ from the blanks, therefore, all alkylammonium cations had to be omitted from the evaluation. However, *n*_w,*i*_ ≅ *n*_tot,*i*_ suggested that the fully cationic chemicals, all of which also had surfactant properties, were not taken up into PDMS. The amounts *n*_PDMS,*i*_ of the fully anionic chemicals in PDMS and the acids at a high pH value were also mostly negligible.

For the remaining 276 chemicals with detections in PDMS and water, only those with a mass balance *n*_tot,*i*_ = 100 ± 50% × *n*_spiked,*i*_ (where *n*_spiked,*i*_ refers to the amount of spiked chemicals) and 0.01 < *n*_PDMS,*i*_/*n*_w,*i*_ < 100 were included in the further evaluation (Figure S2 and Table S3). Mass balance was often much lower than 100% at pH 11.5, pointing to pH-dependent hydrolysis of the test chemicals.

The potential for hydrolysis^39^ was predicted with the Chemical Transformation Simulator (CTS).^40^ Hydrolysis scores in Table S3 varied from scores 6 to 1 from fast to slow hydrolysis.^39^ Hydrolysis pathways with scores <3 should not affect stability in the given setup because the hydrolysis half-life was predicted to be >60 days for these processes (amide hydrolysis, sulfonylurea hydrolysis, thiocarbamate hydrolysis). The chemicals with reduced recovery matched well with the prediction of hydrolysis (Table S3). Hydrolysis that could be relevant for the time frame of the experiments, especially at pH 11.5, include carboxylic acid ester hydrolysis, cyclic urea hydrolysis, lactone hydrolysis, and sulfonylurea hydrolysis. Twenty-six chemicals were excluded, and for additional 26 chemicals, one or two pH values had to be excluded because observed losses appeared to be related to hydrolysis.

After all filtering steps, we were left with 190 chemicals for which the *D*_PDMS/w,*i*_ could be reliably derived, 93 of which were neutral, 38 were monoprotic acids, 42 were monoprotic bases, and 17 were multiprotic substances (Figure S2).

### 3.3 *K*_PDMS/w_ of Neutral Chemicals

The concentrations of chemicals in PDMS (*C*_PDMS,*i*_) and water (*C*_water,*i*_) were generally consistent after 5 and 10 days of exposure and there were no significant time differences (paired *t*-test, *p* = 0.5254, effective pairing with *r* = 0.99), indicating that equilibrium had been reached. Sorption isotherms may be nonlinear, which is more relevant for adsorption, while absorption is rather a concentration-independent partitioning process. The linear sorption isotherms for three illustrative examples (fenofibrate, atrazine, and fluconazole) are depicted in Figure S4.

All valid apparent distribution ratios log*D*_PDMS/w_(pH) of neutral chemicals are listed in Table S4. On the example of five neutral chemicals (fenofibrate, piperonyl butoxide, triclocarban, atrazine, and fluconazole), Figure S5 illustrates that the log*D*_PDMS/w_(pH) did not differ between pH 3, 7.4, and 11.5, and a paired *t*-test of all chemicals at the three pH values confirmed that the difference was not significant (*p* = 0.5, effective pairing: *p* < 0.0001, *r*(pH 3 vs pH 7.4) = 0.98, *r*(pH 7.4 vs pH 11) = 0.99, *r*(pH 3 vs pH 11) = 0.98). This means that the measurements are independent of the pH and the *D*_PDMS/w_(pH) equals to the *K*_PDMS/w_ of the neutral species, which is reported as a mean of the log *D*_PDMS/w_(pH) in Table S5.

**Figure 1:**
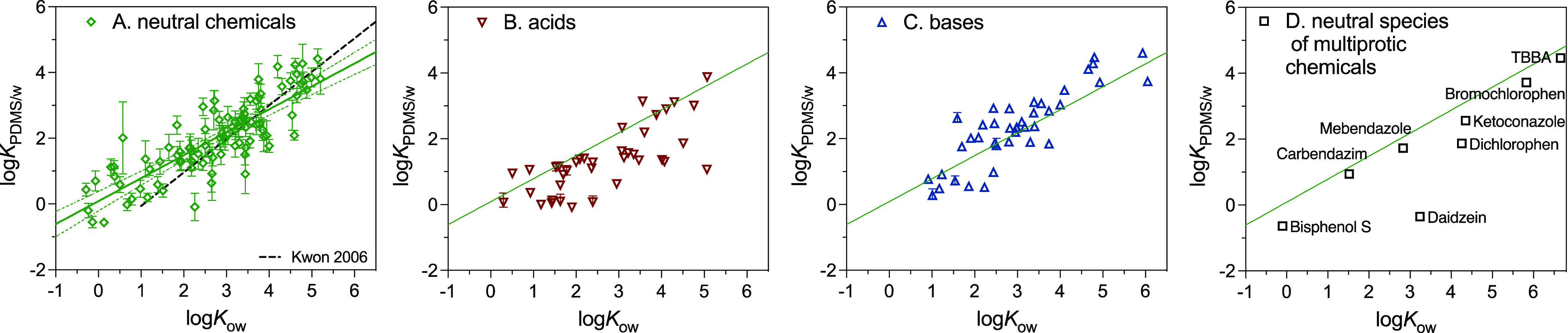
(A) Correlation of octanol–water partition constant (log *K*_ow_) with polydimethylsiloxane (PDMS)–water partition constant (log *K*_PDMS/w_) of neutral chemicals. The green line is the best fit (eq 11, with dotted green confidence interval), the broken black line is the QSAR model from Kwon et al.^41^ The log *K*_PDMS/w_ refers to the mean of values measured at three pH levels and error bars are standard deviations of the mean (Table S5). (B) Log *K*_PDMS/w_ of the neutral species of acids (Table S5), extrapolated from the speciation plots (Figure S7) and error bars corresponding to the 95% confidence interval of the slope of the regression (eq 5). (C) Log *K*_PDMS/w_ of the neutral species of bases (Table S5), extrapolated from the speciation plots (Figure S8) and error bars corresponding to the 95% confidence interval of the slope of the regression (eq 5). (D) Log *K*_PDMS/w_ of the neutral species of the multiprotic chemicals (Table S5). TBBA = tetrabromobisphenol A. (B–D) The green line marks eq 11 derived for neutral chemicals.

There was a linear correlation between the log *K*_ow_ and the log*K*_PDMS/w_ (Figure 1A, green line) described by eq 11. The quantitative structure–activity relationship (QSAR) of this study was similar to but covers a wider log *K*_ow_ range than the QSAR by Kwon et al.,^41^ which is depicted as a broken black line in Figure 1A and had been derived for more hydrophobic chemicals and did not include experimental data at log *K*_ow_ < 1.
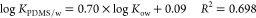
11Evidently, the more hydrophobic an organic chemical is, the longer it takes to reach equilibrium. Kwon et al.^41^ used the same shaking method as the method applied here for chemicals only up to log *K*_ow_ of 4.2 with shaking of up to 3 days but moved onto partition-controlled delivery and a dynamic permeation method for more hydrophobic chemicals. Our study covered a range of log *K*_ow_ up to 5.2 for neutral chemicals and 7.7 for multiprotic IOCs with shaking for 5 and 10 days.

The log *K*_PDMS/w_ of the more hydrophilic neutral chemicals measured in this study were within one log-unit to the experimental log *K*_PDMS/w_ reported in the literature (Table S5 and Figure S6).^2,5,41−51^ The log *K*_PDMS/w_ of more hydrophobic chemicals deviated more than the less hydrophobic chemicals, but the literature data also often covered a very wide range for different experimental methods. As a matter of fact, the good agreement with the QSAR of Kwon et al.,^41^ which was based on experimental data determined with three different methods for different ranges of hydrophobicity, is an indication that our experiments are valid and that the other literature data for these chemicals might need to be further scrutinized.

The log *K*_PDMS/w_ could be derived for 93 neutral chemicals (plus many IOCs as described below) in one mixture. The *m*_PDMS_/*V*_water_ ratios were varied to assure optimal experimental conditions for as many chemicals as possible. However, with hydrophobicity covering six orders of magnitude, there are some limitations for the more hydrophobic chemicals and large uncertainty for the hydrophilic chemicals due to their low concentrations in water or PDMS, respectively, which could only partly be compensated for by the experimental design.

### 3.4 *D*_PDMS/w_(pH) of Monoprotic IOCs

The p*K*_a_ is crucial for understanding the role of speciation in the partitioning of IOCs into PDMS. We measured p*K*_a_ values for six monoprotic IOCs with lacking or inconsistent experimental data from different sources (Table S7). The fractions of different species at pH 3, 7.4, and 11.5 are listed in Table S6 for illustration but in the speciation plots discussed below, we used the pH value measured for each experiment individually.

The *D*_PDMS/w_(pH) were highly dependent on the pH for all IOCs (Table S5). Unfortunately, hydrolysis at pH 11 coincided in several cases with a fully charged form of the organic acids, therefore, we cannot clearly say if the lack of data is caused by a lack of uptake of the charged species into PDMS or other loss processes. The *D*_PDMS/w_(pH) was plotted against the fraction of neutral species α_neutral_ in Figure S7 for 38 monoprotic acids and Figure S8 for 40 monoprotic bases. Atenolol and metoprolol could not be plotted because too many data points were invalid due to *n*_PDMS,*i*_/*n*_w,*i*_ < 0.01. For most acids and bases, the intercept of the linear regression (eq 5) was crossing the intercept at zero, which means that *K*_PDMS/w,charged species_ would be negligible.

With the intercept being zero for almost all monoprotic acids and bases, the *K*_PDMS/w_ of the neutral species (Table S5) could be derived directly from the slope of the linear regression of eq 5 (Figures S7 and S8). The thus extrapolated *K*_PDMS/w_ of the neutral species for the acids (Figure 1B) and bases (Figure 1C) aligned generally with the QSAR equation derived with neutral chemicals. In case of the acids, the extrapolated *K*_PDMS/w_ of the neutral species were often lower than the QSAR.

The negligible uptake of charged species of IOCs has not yet been reported for PDMS but has been established for polyacrylate (PA),^52−54^ PE,^55^ and PS.^26^ Several studies have reported experimental log*D*_PDMS/w_ of organic acids and bases at pH between 3 and 7.8 (Table S5^2,5,42−48,50,51^), and many of these studies have not even reported the pH and none evaluated the pH dependence of partitioning. Consequently, there was no good agreement between the data reported in the literature and the *D*_PDMS/w_(pH 7.4) in the present study (Figure S9).

### 3.5 *D*_PDMS/w_(pH) of Multiprotic IOCs

The log*D*_PDMS/w_(pH) of 17 multiprotic chemicals are listed in Table S5 and their p*K*_a_ values and speciation are shown in Table S6. We experimentally quantified p*K*_a_ values for 16 complex multiprotic IOCs, for which only predicted values were available, and verified the p*K*_a_ value of labetalol^56^ (Table S7).

There was no extrapolation to the neutral species possible. The log*K*_PDMS/w_ of those multiprotic chemicals that were predominantly neutral at a given pH are plotted in Figure 1D as a function of log *K*_ow_ and, with exception of daidzein, they agreed well with the QSAR derived for neutral chemicals (eq 11).

**Figure 2:**
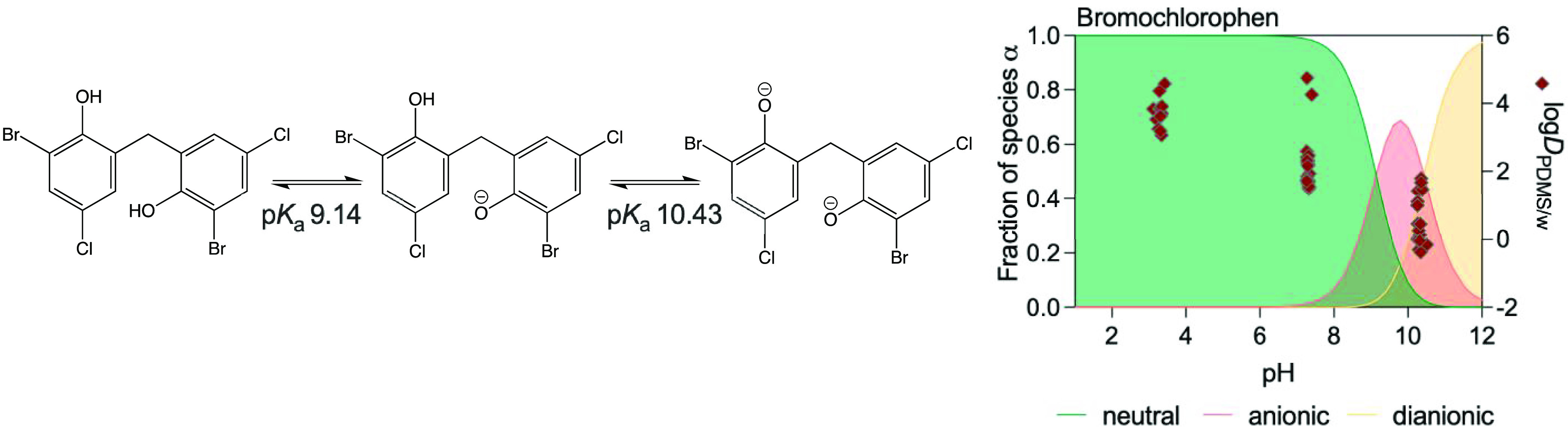
Fractions of all relevant species α_*j*_ (calculated with eqs 8–10) of the diprotic acid bromochlorophen as a function of pH (left *y*-axis, colored areas, green: neutral species, red: anionic species, and yellow: dianionic species) and the measured polydimethylsiloxane–water distribution ratios (log *D*_PDMS/w_, Table S4) (right *y*-axis, diamond symbols). The p*K*_a_ values were measured with the UV-metric method using Sirius T3 (Table S7).

**Figure 3:**
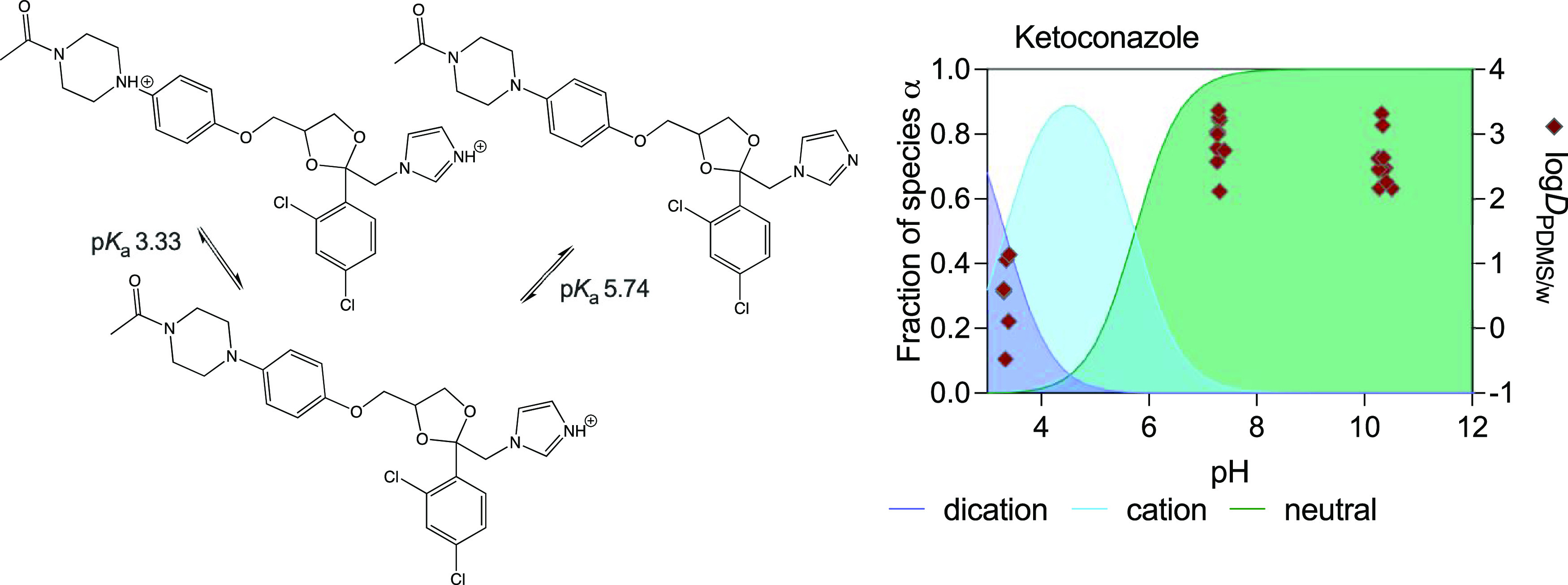
Fractions of all relevant species α*_j_* (calculated with eqs 8–10) of the diprotic base ketoconazole as a function of pH (left *y*-axis, colored areas: violet: dication, blue; cation, green: neutral species) and the measured polydimethylsiloxane–water distribution ratios (log* D*_PDMS/w_, Table S4) (right *y*-axis, diamond symbols). The p*K*_a_ values were measured with the UV-metric method using the Sirius T3 (Table S7).

**Figure 4:**
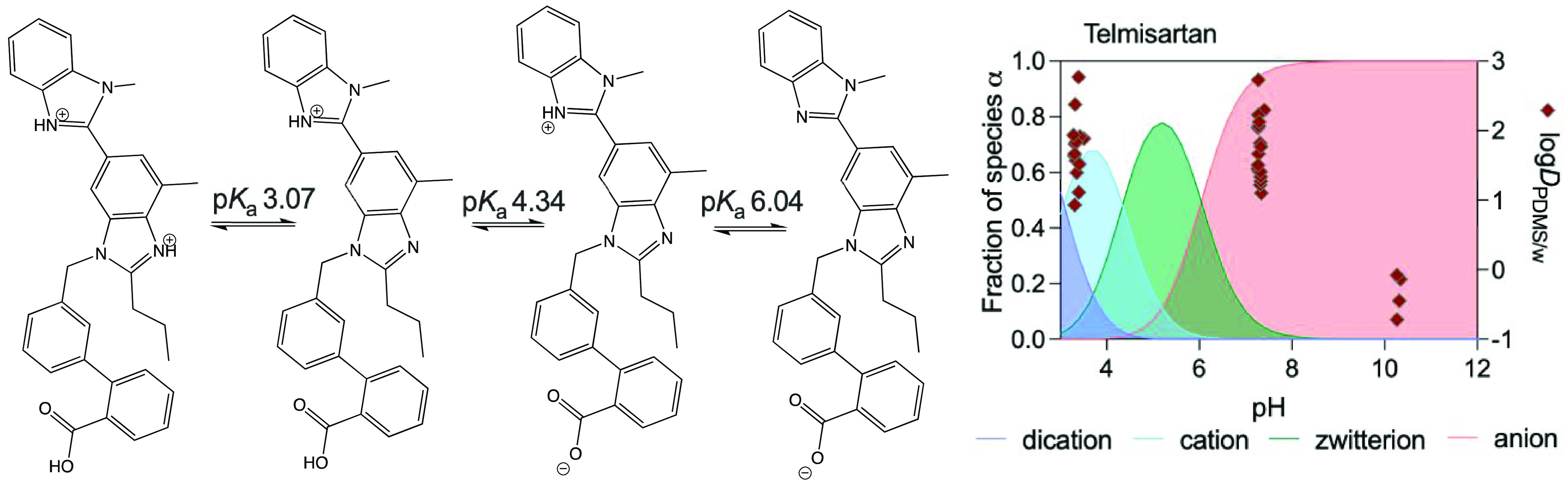
Fractions of all relevant species α*_j_* of telmisartan, a triprotic IOC with one acidic and two basic functional groups as a function of pH (left *y*-axis, colored areas: violet: dication, blue: cation, green: neutral species, red: anionic species) and the measured polydimethylsiloxane–water distribution ratios (log* D*_PDMS/w_, Table S4) (right *y*-axis, diamond symbols). The p*K*_a_ values were measured in this study (Table S7). The fractions of the four species were calculated with the equations given in the Appendix of Escher et al.^20^

For visualization of the speciation dependence, we also plotted all log *D*_PDMS/w_(pH) as a function of pH as red diamonds in Figures 2–4 and S10–S12, overlaying speciation plots, where the fractions of the different species were indicated by colored areas.

All of the diprotic acids tetrabromobisphenol A, bromochlorophen, dichlorphen, daidzein, and bisphenol S (Figure 2 and S10) had substantially higher *D*_PDMS/w_ at the pH values, where they were neutral, but they were still detectable when virtually no neutral species was present anymore. This is shown in Figure 2 for bromochlorophen that has 1000-fold lower *D*_PDMS/w_ in its approximately 50% anionic and 50% dianionic forms at pH 10.25–11.00 than in its neutral form at pH 3 but the partitioning of the charged species was not fully negligible. The diprotic acid and metolachlor metabolite CGA 357704 showed an unexpected pH dependence in Figure S10 with the dianionic form having a ten times higher *D*_PDMS/w_ than the anion, which could potentially be explained by a strong chelate-type complex formation of the dianion with cations.

The diprotic base ketoconazole (Figure 3) mirrored the behavior of the diprotic acids with *D*_PDMS/w_ of the neutral species at pH 11 being 300 times higher than that of the dicationic species at pH 3.

IOCs with acid and base functions such as pioglitazone, labetalol, mebendazole, carbendazim, 2-aminobenzimidazole, quinmerac, and clothiadin differed widely in their speciation (Table S4), but, generally, the neutral species also showed the highest *D*_PDMS/w_ (Table S5 and Figure S11). This was also the case for triprotic acids with two acidic and one basic functional groups, levothyroxine and candesartan (Table S5 and Figure S12). The dianionic forms of levothyroxine and candesartan were not detected in PDMS. Telmisartan is a triprotic IOC with one acidic and two basic functional groups. Anions and cations of telmisartan had similar *D*_PDMS/w_, and the neutral species was not relevant at the measured pH values (Table S5 and Figure 4). It is interesting to note that the bulky multiprotic compounds often had rather substantial *D*_PDMS/w_ even in their charged forms, which might be explained by charge delocalization and conformations that shield the charge.

Such an effect seems to be more important than the initially postulated ion pairing. The mixture of 514 chemicals that contain many cations and anions of highly variable structures and hydrophobicity seems not to increase the uptake of individual charged organics as shown by the negligible uptake of many simple monoprotic acids. This is substantiated by the good agreement of the extrapolated *K*_PDMS/w_ of the neutral species with the QSAR shown in Figure 1 that does not indicate that there are any mixture effects. In contrast, bulky ions in very hydrophobic core molecules appear to be favorable for the uptake into PDMS.

## 4 Implications for Environmental Research

### 4.1 High-Throughput Methods for Quantification of Basic Physicochemical Properties

This study is a demonstration of how advances in chemical analysis allow us to take new pathways to quantify basic physicochemical properties. With the advanced capabilities of target analysis that allows the simultaneous quantification of hundreds of compounds, we are able to quantify the *K*_PDMS/w_ of 190 chemicals in a mixture but we can envisage these methods to be expanded to suspect and nontarget screening using peak ratios.^57,58^

By comparing with experimental data that had been obtained with single chemicals or mixtures of a few components, we were able to demonstrate that such measurements are reliable for multicomponent mixtures. We performed 65 different experiments with this mixture, covering a range of *m*_PDMS_/*V*_w_ ratios from 0.01 to 100 and spiked concentrations from 1 to 50 μg/mL for each mixture component. This is still less tedious than running 190 chemicals individually in multiple replicates that would require easily >1000 experiments.

This was a feasibility study and targeted to deliver experimental data for our work with ex situ sediment and suspended particulate matter passive sampling^8,10,11,59^ but the principle could be extended to the experimental determination of a wide range of polymer–water partition constants. By grouping chemicals in classes of similar hydrophobicity, one could further expand the range of reliably determined *D*_PDMS/w_(pH).

As a matter of fact, one working hypothesis in this endeavor was that the complexity of the mixture might allow ion pairing and therefore enhanced uptake of charged chemicals into PDMS as is the case for octanol–water partitioning.^60−62^ In realistic environmental mixtures, such interactions might be possible. We did indeed detect very low levels of ionic chemicals, in particular, hydrophobic ions, in PDMS, but the *D*_PDMS/w_(pH) could not be quantified with high precision and was also by at least three orders of magnitude lower than the prediction for the corresponding neutral species. If they have any role, the role of ion pairs taken up into PDMS is minor to negligible.

In the era of big data, we are inclined to rely on predictive models for physicochemical properties, but, unfortunately, models are often abused to make prediction outside the domain of applicability. Therefore, it is important to increase the number of experimental data in and outside the applicability domain, which we did here for hydrophilic chemicals and IOCs, and we also expanded the database of neutral chemicals to lower hydrophobicity. It is often considered that silicone is suitable as a sampler for hydrophobic chemicals only but silicone does sample hydrophilic neutral chemicals as we have clearly demonstrated here.

The issue of lack of experimental data concerns especially multiprotic IOCs despite their presence in aquatic systems. For example, telmisartan has been identified as a priority pollutant and mixture risk driver in European surface waters^63^ but this complex molecule had no published experimental p*K*_a_. Only predictions were available. Our experiments confirmed most predictions but the subtle differences around the ambient pH levels might matter. For instance, the p*K*_a_ value of triclosan was 8.8 predicted with ACD pKa/GALAS,^64^ 8.1 according to experiments measured with an unreported method,^65,66^ and 7.9 according to the Merck index,^67^ and our newly measured p*K*_a_ of 8.11 lay in the middle (Table S7). For tetrabromobisphenol A, the measured p*K*_a_ values were substantially lower than the p*K*_a_ predicted by ACD pKa/GALAS^64^ (Table S7), which means that a higher fraction of tetrabromobisphenol A was double-deprotonated at typical environmental conditions.

### 4.2 Implications for Sampling of Mixtures under Field Conditions

Underestimation of the role of hydrophilic organics and IOCs might not pose any problems when using chemical analysis to quantify chemicals in polymer extracts from *ex situ* passive sampling of sediments and biota. The ionization-corrected *D*_PDMS/w_(pH) can be used for conversion from PDMS to aqueous concentrations, but it is not clear if there are hydrophobicity- and ionization-independent PDMS–sediment distribution ratio log *D*_PDMS/sed_ and PDMS–tissue distribution ratio log *D*_PDMS/tissue_ as for neutral hydrophobic chemicals.

If polymer-based passive samplers are used in combination with bioassays,^7,9,68^ one needs to keep in mind that hydrophilic organics and the neutral species of IOCs are taken up to different degrees and may contribute to the mixture effects but it is not possible to assign a robust distribution ratio independent on the mixture components to bioanalytical equivalent concentrations. Typically, the potency of hydrophilic organics and IOCs is lower than that of hydrophobic organic chemicals but some might exhibit a specific mode of action, e.g., many weak organic acids are potent uncouplers of oxidative phophorylation.^20^ The issue of overlooking IOCs might be exacerbated if silicone rubber samplers are used *in situ* for sampling water directly in the field, where, in addition to the speciation, differences in uptake kinetics^69^ have to be considered.

### 4.3 Implications for (Micro)plastic Research

The present study also has important implications for (micro)plastic research. Binding of organic contaminants to microplastic is a highly contested topic. Microplastic may serve as a vector for organic micropollutants, and while the initial focus was on hydrophobic chemicals and persistent organic pollutants, studies on this topic have exploded and expanded the chemicals to polar hydrophilic and ionizable chemicals.^23^ A thorough study evaluated the pH dependence of sorption of neutral and ionic organic compounds to polyethylene and polystyrene microparticles of <1 mm and concluded that the contribution of the charged species to sorption was negligible.^26^ Our results confirmed this finding for PDMS. Another study^70^ evaluated the pH dependence of the sorption of triclosan at pH values near the p*K*_a_ but did not even consider the possibility of partitioning/absorption of the neutral species as compared to adsorption, which may have well played a role at pH values, where the neutral species dominated. Although the kinetics of partitioning to the rubbery polymer PDMS is faster than that to the glassy polymers PE,^71,72^ PS,^73^ and PVC,^74^ the method developed in this study may serve as an experimental model to explore the difference between absorption and adsorption in the research of the role of (micro)plastic as a vector for organic micropollutants.

## Supporting Information Available

Additional information on spectrometric pH determination; experimental flow chart; changes of pH during the experiment, comparison of *D*_PDMS/w_(pH) under different conditions; comparison of results with literature data; *D*_PDMS/w_(pH) vs speciation plots for acids and bases; detailed description of speciation of the multiprotic chemicals (PDF)
Excel file with chemical descriptors, concentrations of spiked chemicals; practical mass of polydimethylsiloxane and volumes of water used for the partitioning experiment at three pH values; detected amounts in PDMS and water; mass balance; predicted hydrolysis; and final valid *D*_PDMS/w_(pH), derived *K*_PDMS/w_ of the neutral species, and predicted and measured acidity constants (XLSX)
