# Supplementary material for: pH-Dependent Partitioning of Ionizable Organic Chemicals between the Silicone Polymer Polydimethylsiloxane (PDMS) and Water
Source: ACS Environ Au. 2022 Feb 16;2(3):253–62. doi: 10.1021/acsenvironau.1c00056 (PMC10114720; doi:10.1021/acsenvironau.1c00056)
Supplement: Supplementary file 1 — vg1c00056_si_001.pdf [file vg1c00056_si_001.pdf]

pH-dependent partitioning of ionizable organic chemicals between the silicone polymer polydimethylsiloxane (PDMS) and water

Lili Niu[a,b], Luise Henneberger[a], Julia Huchthausen[a], Martin Krauss[c], Audrey Ogefere[a] and Beate I. Escher[a,d]*

[a]*UFZ – Helmholtz Centre for Environmental Research, Department of Cell Toxicology, 04318 Leipzig, Germany*

[b]*Key Laboratory of Pollution Exposure and Health Intervention of Zhejiang Province, Interdisciplinary Research Academy (IRA), Zhejiang Shuren University, Hangzhou 310015, China*

[c]*Helmholtz Centre for Environmental Research, Department of Effect Directed Analysis, 04318 Leipzig, Germany*

[d]*Eberhard Karls University of Tübingen, Center for Applied Geoscience, Schnarrenbergstr. 94-96, 72076 Tübingen, Germany*
*Email: beate.escher@ufz.de

Table of content

Figure S1. Absorbance and calibration regressions used for pH determination.

Figure S2. Flow chart for selecting chemicals with valid polydimethylsiloxane-water distribution ratios $D_{PDMS/w.}$

Figure S3. Changes of pH in the water phase spiked with chemicals before and after the experiment.

Figure S4. Sorption isotherms for three illustrative examples: A. fenofibrate, B. atrazine, C. fluconazole.

Figure S5. Comparison of the polydimethylsiloxane-water distribution ratios ($D_{PDMS/w}$) of neutral chemicals.

Figure S6. Comparison of the mean polydimethylsiloxane-water partition constants ($K_{PDMS/w}$) of neutral chemicals with published literature data.

Figure S7. The measured polydimethylsiloxane-water distribution ratios ($D_{PDMS/w}$) of monoprotic acids as a function of their neutral fractions ($\alpha_{neutral}$) at different pH levels.

Figure S8. The measured polydimethylsiloxane-water distribution ratios ($D_{PDMS/w}$) of monoprotic bases as a function of their neutral fractions ($\alpha_{neutral}$) at different pH levels.

Figure S9. Comparison of mean of the polydimethylsiloxane-water partition constant ($K_{PDMS/w}$) of the neutral species of acids and bases with published literature data.

Figure S10. The fractions of all relevant species α_i of the diprotic acids as a function of pH and the measured polydimethylsiloxane-water distribution ratios.

Figure S11. The fractions of all relevant species α_i of diprotic acid/bases as a function of pH and the measured polydimethylsiloxane-water distribution ratios (log$D_{PDMS/w}$).

Figure S12. The fractions of all relevant species α_i of levothyroxine, two triprotic IOC (ionizable organic chemical) with two acidic and one basic functional groups as a function of pH and the measured polydimethylsiloxane-water distribution ratios (log$D_{PDMS/w}$).

The following Tables are in a separate Excel file:

Table S1. Information of chemicals spiked and analyzed in this study (in alphabetical order).

Table S2. Concentrations of spiked chemicals (C_{spiked}), practical mass of polydimethylsiloxane (mPDMS) and volume of water (V_w) used for the partitioning experiment at three pH-values. The masses of chemicals detected in polydimethylsiloxane (n_{PDMS}, ng) and in the water phase (n_w, ng) after blank correction.

Table S3. Mass balance expressed as n_{tot}/n_{spiked} and predicted hydrolysis using the Chemical Transformation Simulator CTS.

Table S4. Experimental conditions for the 65 experiments: volume water (Vw, mL), mass of PDMS (m_{PDMS}, mg), spiked water concentration for each chemical (C_{spiked}, ng/mL) and incubation time. Resulting apparent distribution ratios of chemicals between polydimethylsiloxane and water ($D_{PDMS/w}$(pH)).

Table S5. Partition of neutral chemicals and ionizable organic chemicals (IOC) between polydimethylsiloxane (PDMS) and water: experimental results of this study and comparison with literature.

Table S6. Relevant acidity constants pK_a and the fractions of different species at pH 3, 7.4 and 11.5.

Table S7. Experimentally determined acidity constants pK_a and method used for the determination, literature pK_a.

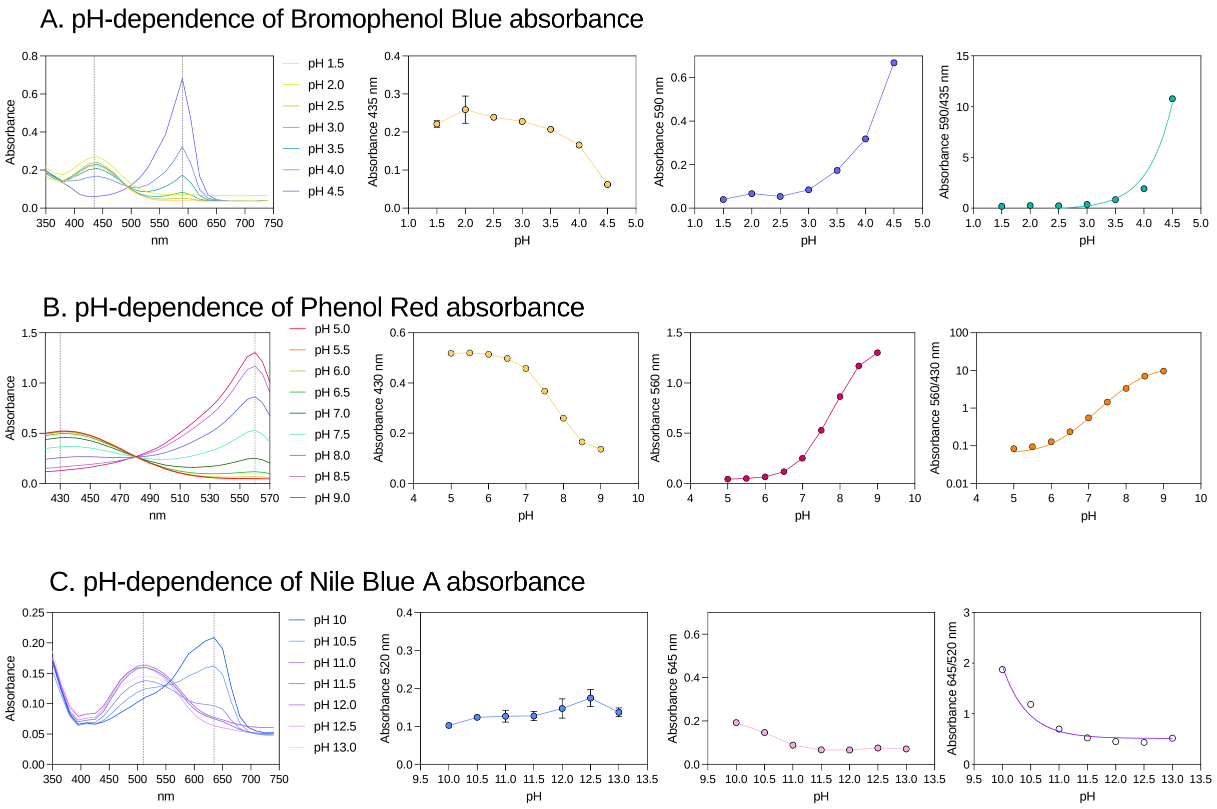

Figure S1. Absorbance and calibration regressions used for pH determination using (A) bromophenol blue, (B) phenol red and (C) Nile blue A.

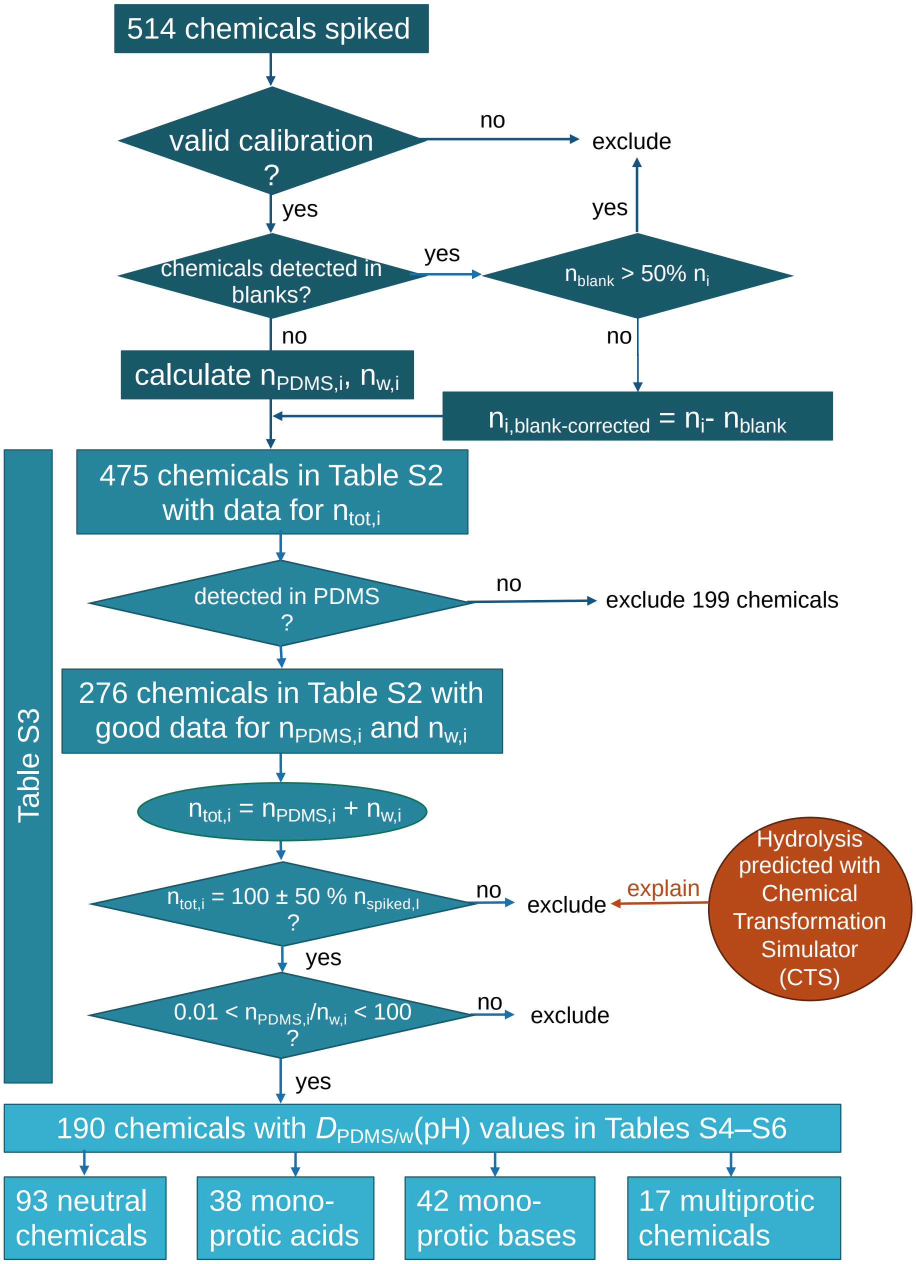

Figure S2. Flow chart for selecting chemicals with valid polydimethylsiloxane-water distribution ratios $D_{PDMS/w}$.

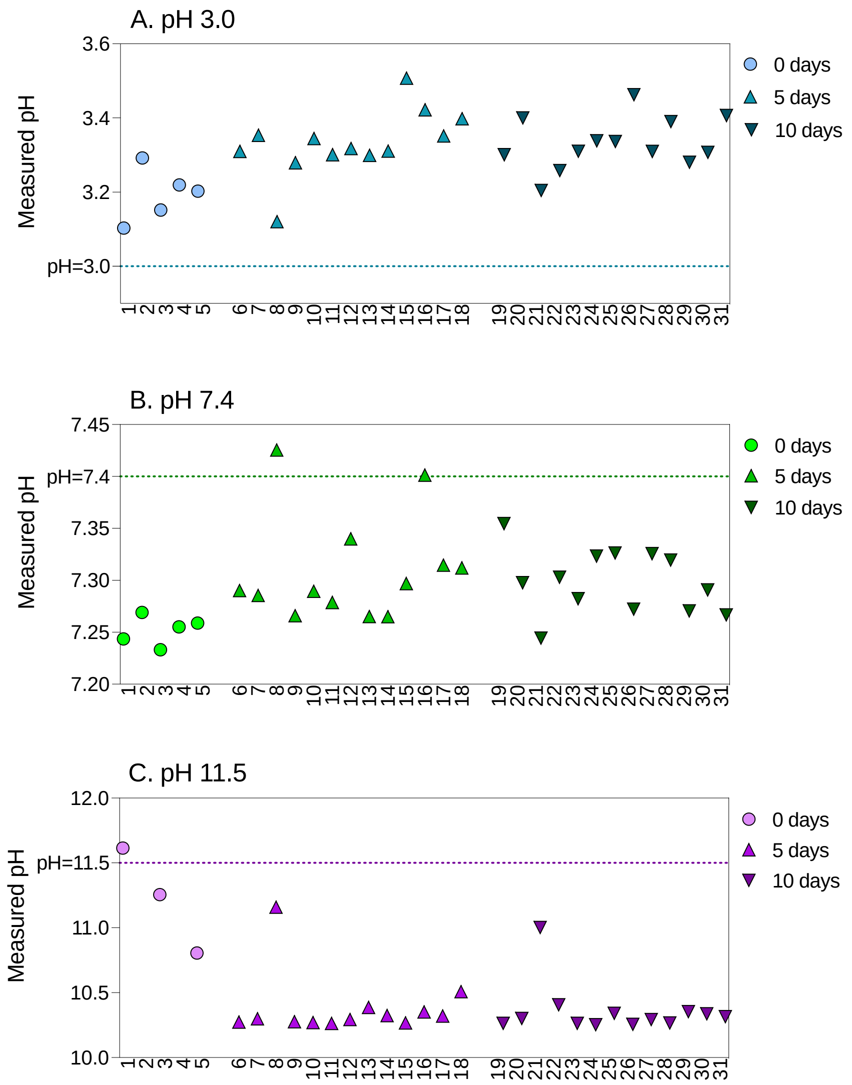

Figure S3. Changes of pH in the water phase spiked with chemicals before and after the experiment: (1)-(5) pH measured directly after spiking (day 0), (6)-(18) after 5 days of incubation and (19)-(31) after 10 days of incubation. (1): blank; (2): 1 ng/mL; (3): 5 ng/mL; (4): 10 ng/mL; (5): 50 ng/mL; (6) and (19): blank with PDMS/w ratio (kg/L) of 1:100; (7) and (20): blank with PDMS/w ratio (kg/L) of 1:10; (8) and (21): 1 ng/mL with PDMS/w ratio (kg/L) of 1:100000; (9) and (22): 5 ng/mL with PDMS/w ratio (kg/L) of 1:10000; (10) and (23): 5 ng/mL with PDMS/w ratio (kg/L) of 1:1000; (11) and (24): 5 ng/mL with PDMS/w ratio (kg/L) of 1:100; (12) and (25): 5 ng/mL with PDMS/w ratio (kg/L) of 1:10; (13) and (26): 10 ng/mL with PDMS/w ratio (kg/L) of 1:1000; (14) and (27): 10 ng/mL with PDMS/w ratio (kg/L) of 1:100; (15) and (28): 10 ng/mL with PDMS/w ratio (kg/L) of 1:10; (16) and (29): 50 ng/mL with PDMS/w ratio (kg/L) of 1:1000; (17) and (30): 50 ng/mL with PDMS/w ratio (kg/L) of 1:100; (18) and (31): 50 ng/mL with PDMS/w ratio (kg/L) of 1:10.

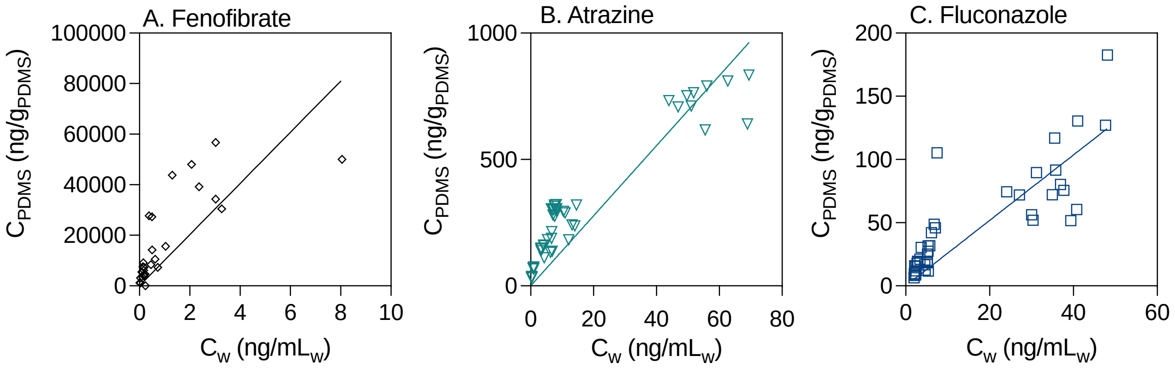

Figure S4. Sorption isotherms for three illustrative examples: A. fenofibrate, B. atrazine, C. fluconazole.

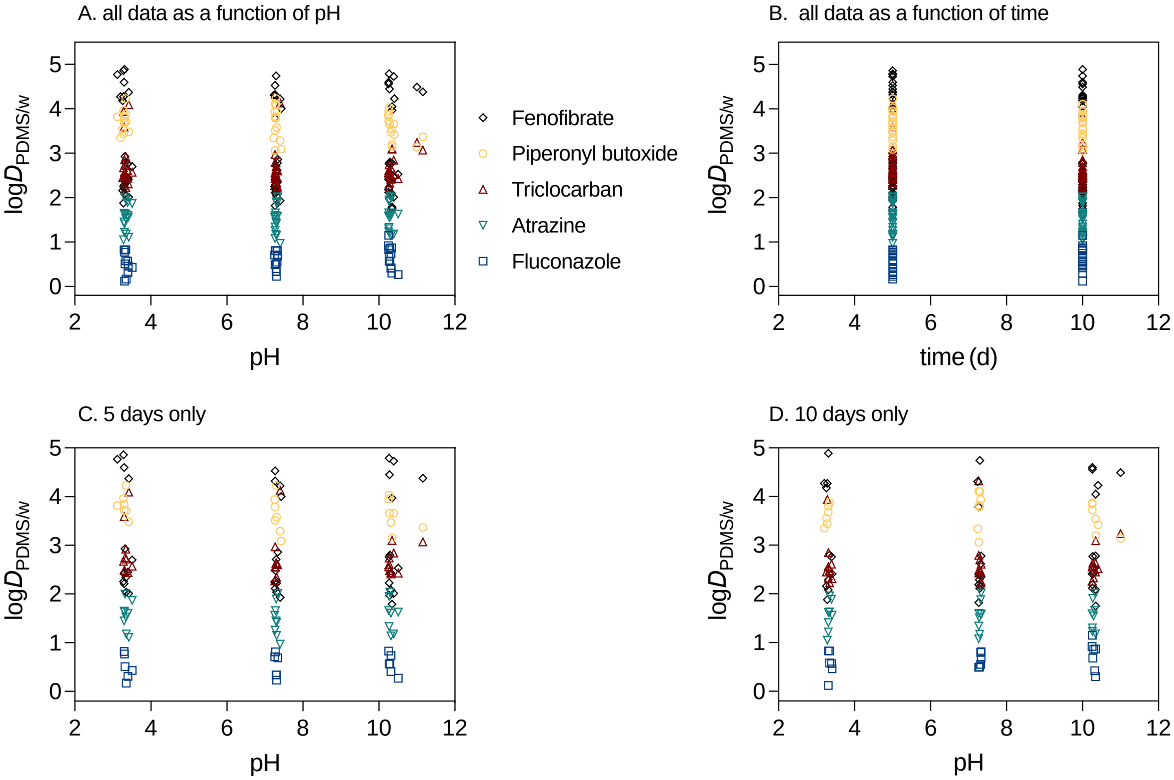

Figure S5. Comparison of the polydimethylsiloxane-water distribution ratios ($D_{PDMS/w}$) of neutral chemicals (A) at different pH-values, (B) all data as a function of time, (C) after 5 days of incubation and (D) after 10 days of incubation on the example of 5 chemicals of different hydrophobicity.

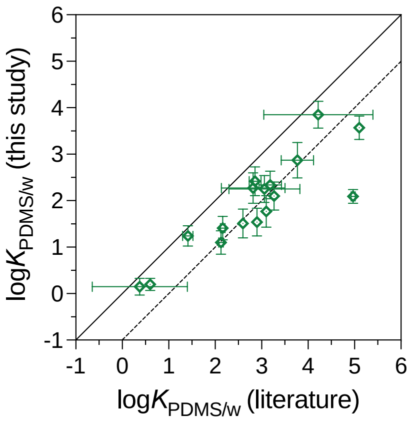

Figure S6. Comparison of the mean polydimethylsiloxane-water partition constants ($K_{PDMS/w}$) of neutral chemicals with published literature data.[1-13] All data in Table S5.

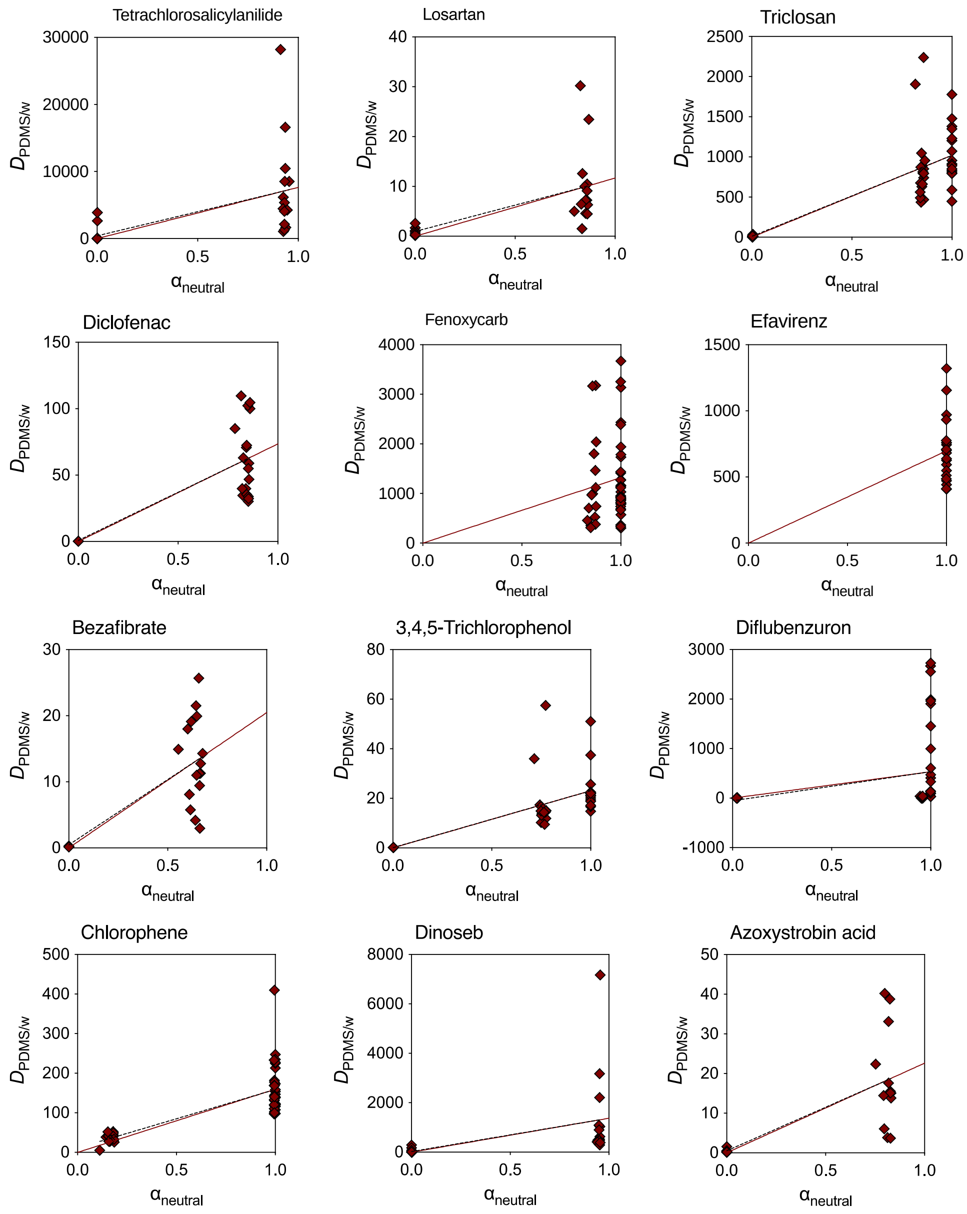

Figure S7. The measured polydimethylsiloxane-water distribution ratios ($D_{PDMS/w}$) of monoprotic acids as a function of their neutral fractions ($\alpha_{neutral}$) at different pH levels. The red line is the best fit with an intercept of 0, the broken black line is the linear regression with the intercept as fit parameter (not in all cases possible). The chemicals are sorted from high hydrophobicity to low hydrophobicity (expressed as logK_{ow} of the neutral species).

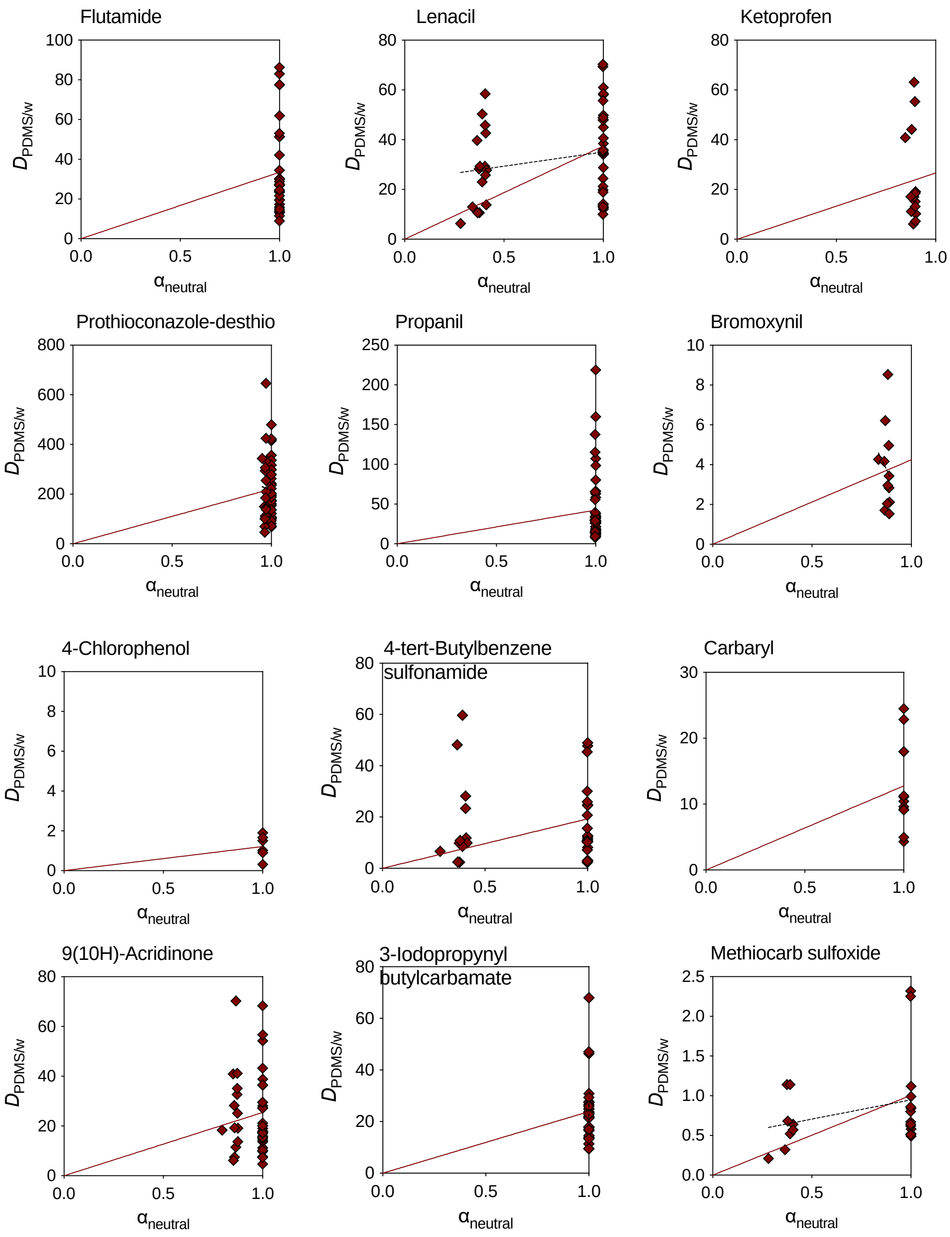

Figure S7 continued. The measured polydimethylsiloxane-water distribution ratios ($D_{PDMS/w}$) of monoprotic acids as a function of their neutral fractions ($\alpha_{neutral}$) at different pH levels. The red line is the best fit with an intercept of 0, the broken black line is the linear regression with the intercept as fit parameter (not in all cases possible). The chemicals are sorted from high hydrophobicity to low hydrophobicity (expressed as logK_{ow} of the neutral species).

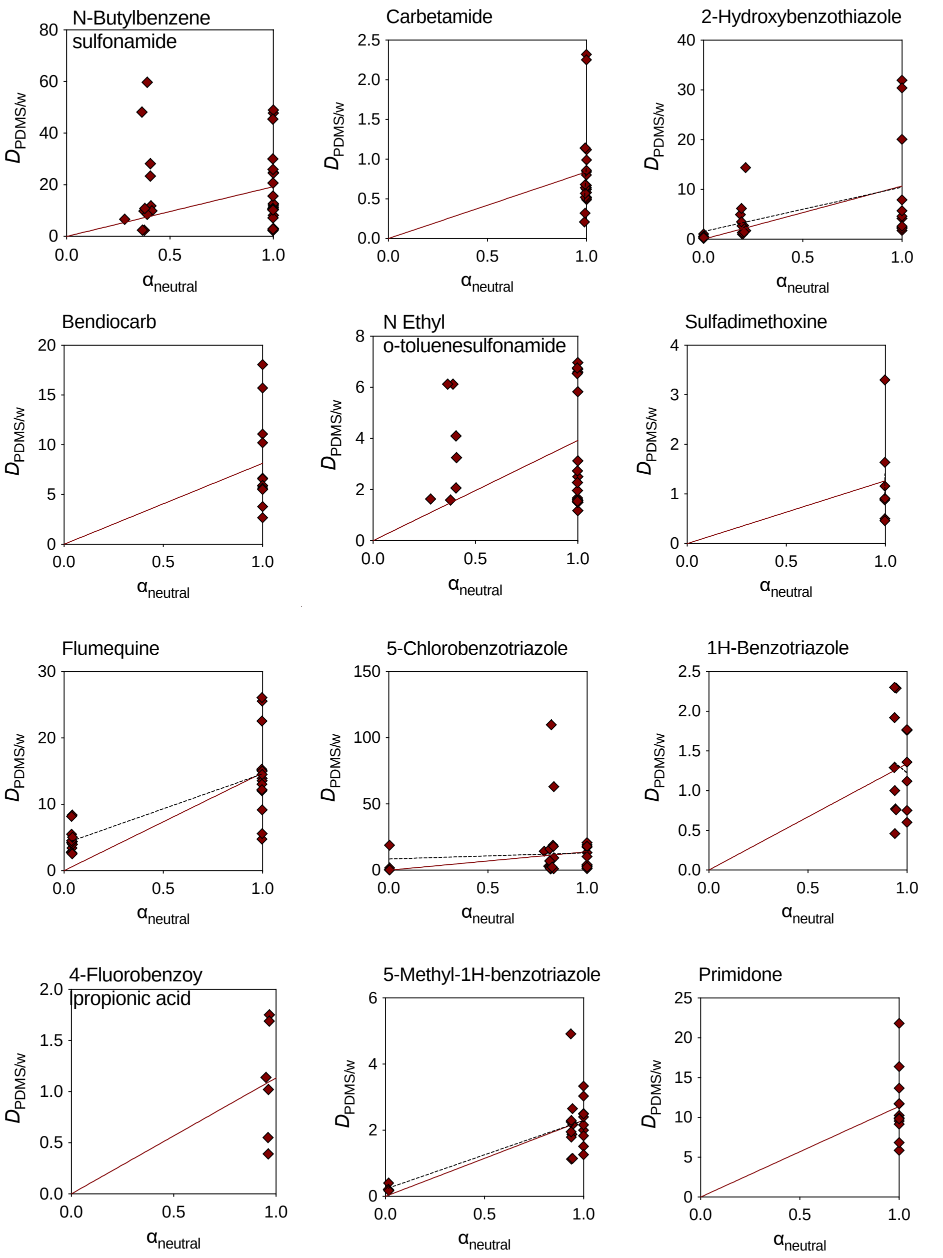

Figure S7 continued. The measured polydimethylsiloxane-water distribution ratios ($D_{PDMS/w}$) of monoprotic acids as a function of their neutral fractions ($\alpha_{neutral}$) at different pH levels. The red line is the best fit with an intercept of 0, the broken black line is the linear regression with the intercept as fit parameter (not in all cases possible). The chemicals are sorted from high hydrophobicity to low hydrophobicity (expressed as logK_{ow} of the neutral species).

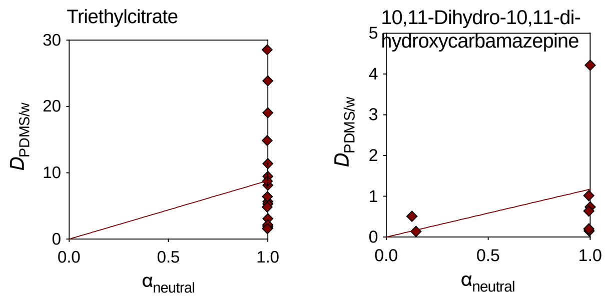

Figure S7 continued. The measured polydimethylsiloxane-water distribution ratios ($D_{PDMS/w}$) of monoprotic acids as a function of their neutral fractions ($\alpha_{neutral}$) at different pH levels. The red line is the best fit with an intercept of 0, the broken black line is the linear regression with the intercept as fit parameter (not in all cases possible). The chemicals are sorted from high hydrophobicity to low hydrophobicity (expressed as logK_{ow} of the neutral species).

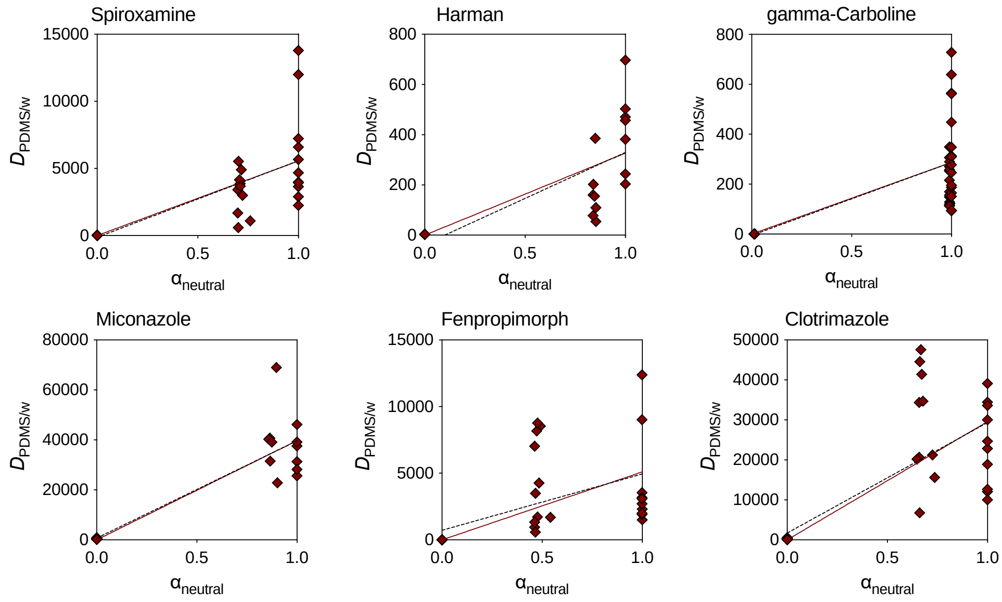

Figure S8. The measured polydimethylsiloxane-water distribution ratios ($D_{PDMS/w}$) of monoprotic bases as a function of their neutral fractions ($\alpha_{neutral}$) at different pH levels. The red line is the best fit with an intercept of 0, the broken black line is the linear regression with the intercept as fit parameter (not in all cases possible). The based are sorted from high hydrophobicity to low hydrophobicity (expressed as logK_{ow} of the neutral species).

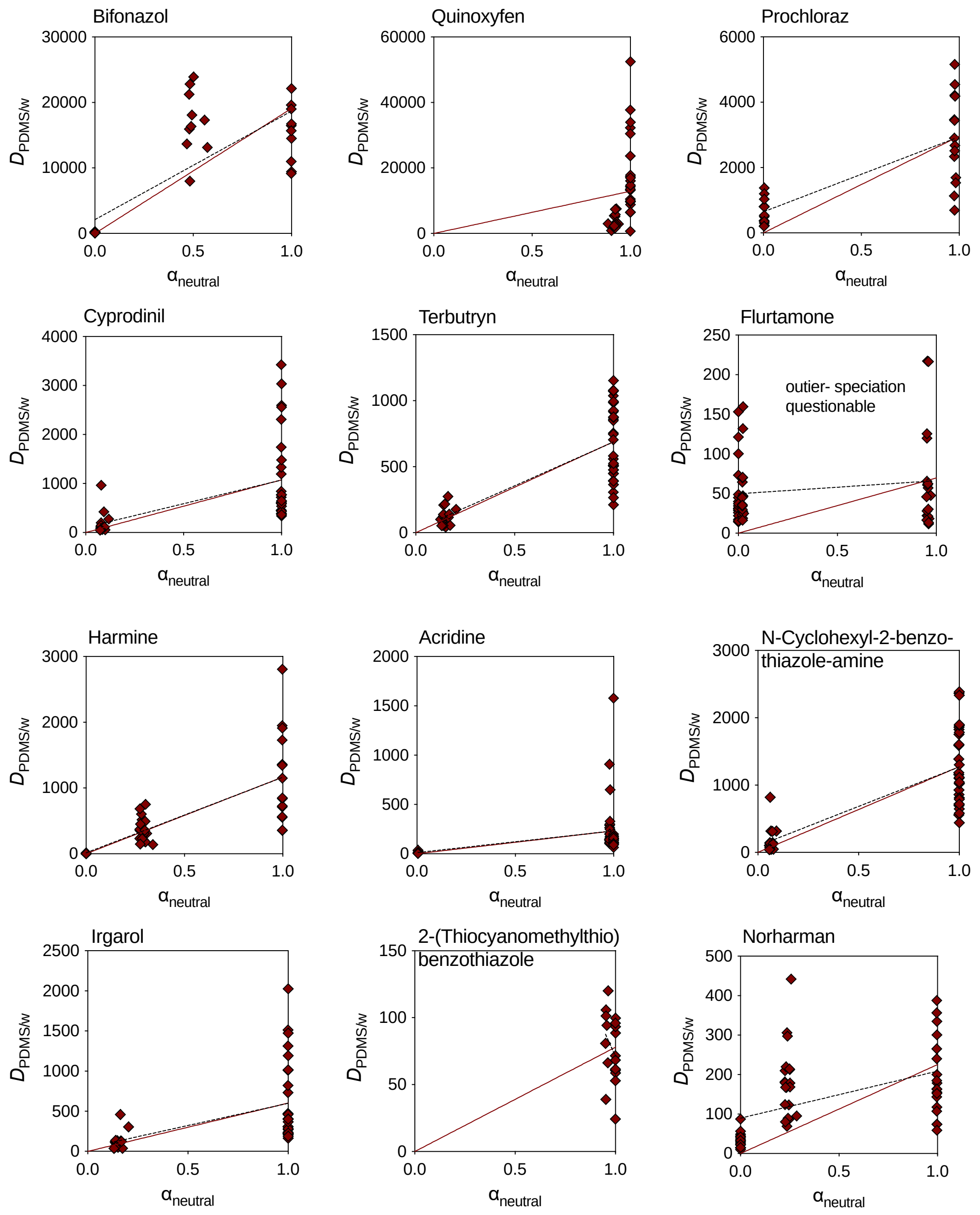

Figure S8 continued. The measured polydimethylsiloxane-water distribution ratios ($D_{PDMS/w}$) of monoprotic bases as a function of their neutral fractions ($\alpha_{neutral}$) at different pH levels. The red line is the best fit with an intercept of 0, the broken black line is the linear regression with the intercept as fit parameter (not in all cases possible). The based are sorted from high hydrophobicity to low hydrophobicity (expressed as logK_{ow} of the neutral species).

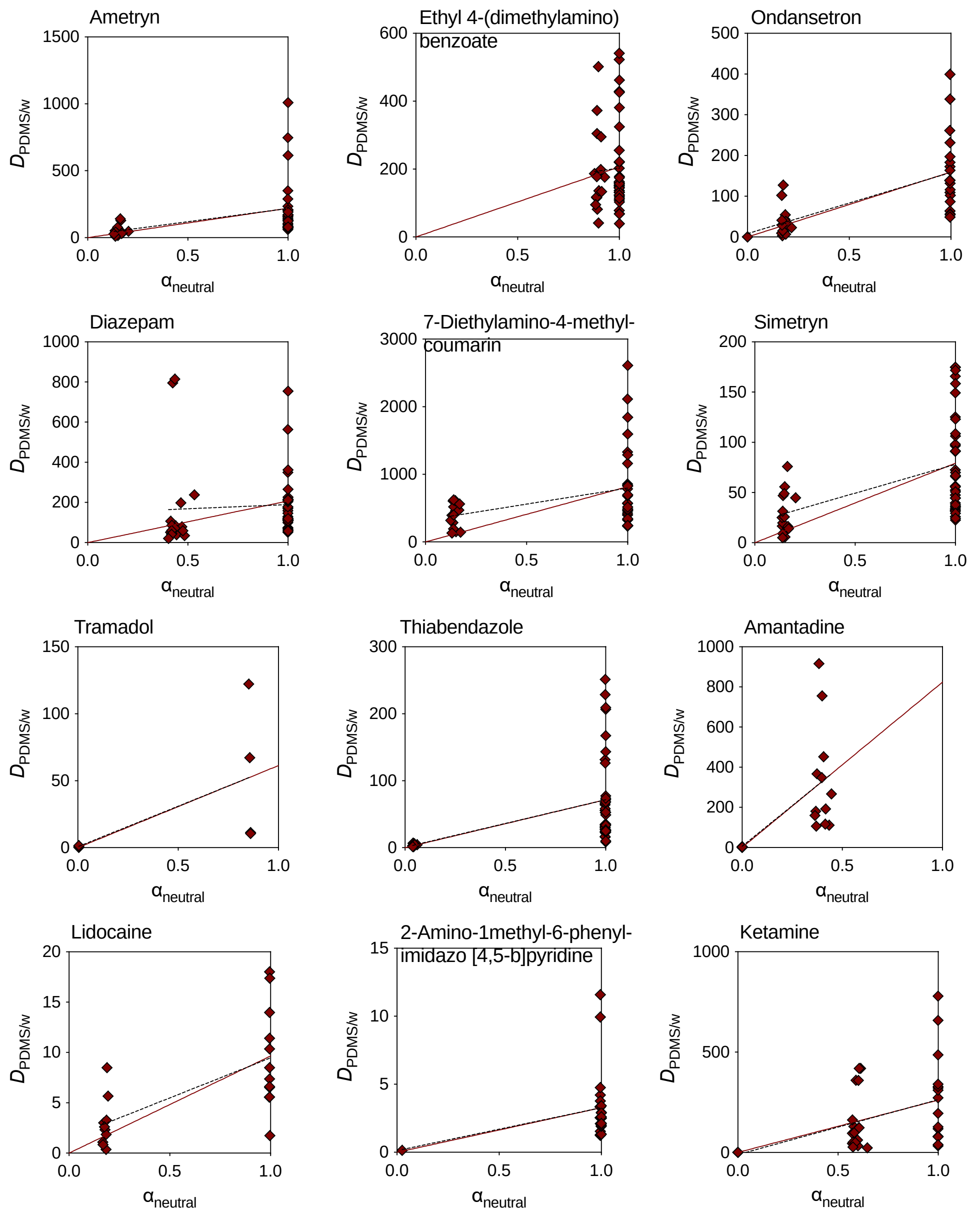

Figure S8 continued. The measured polydimethylsiloxane-water distribution ratios ($D_{PDMS/w}$) of monoprotic bases as a function of their neutral fractions ($\alpha_{neutral}$) at different pH levels. The red line is the best fit with an intercept of 0, the broken black line is the linear regression with the intercept as fit parameter (not in all cases possible). The based are sorted from high hydrophobicity to low hydrophobicity (expressed as logK_{ow} of the neutral species).

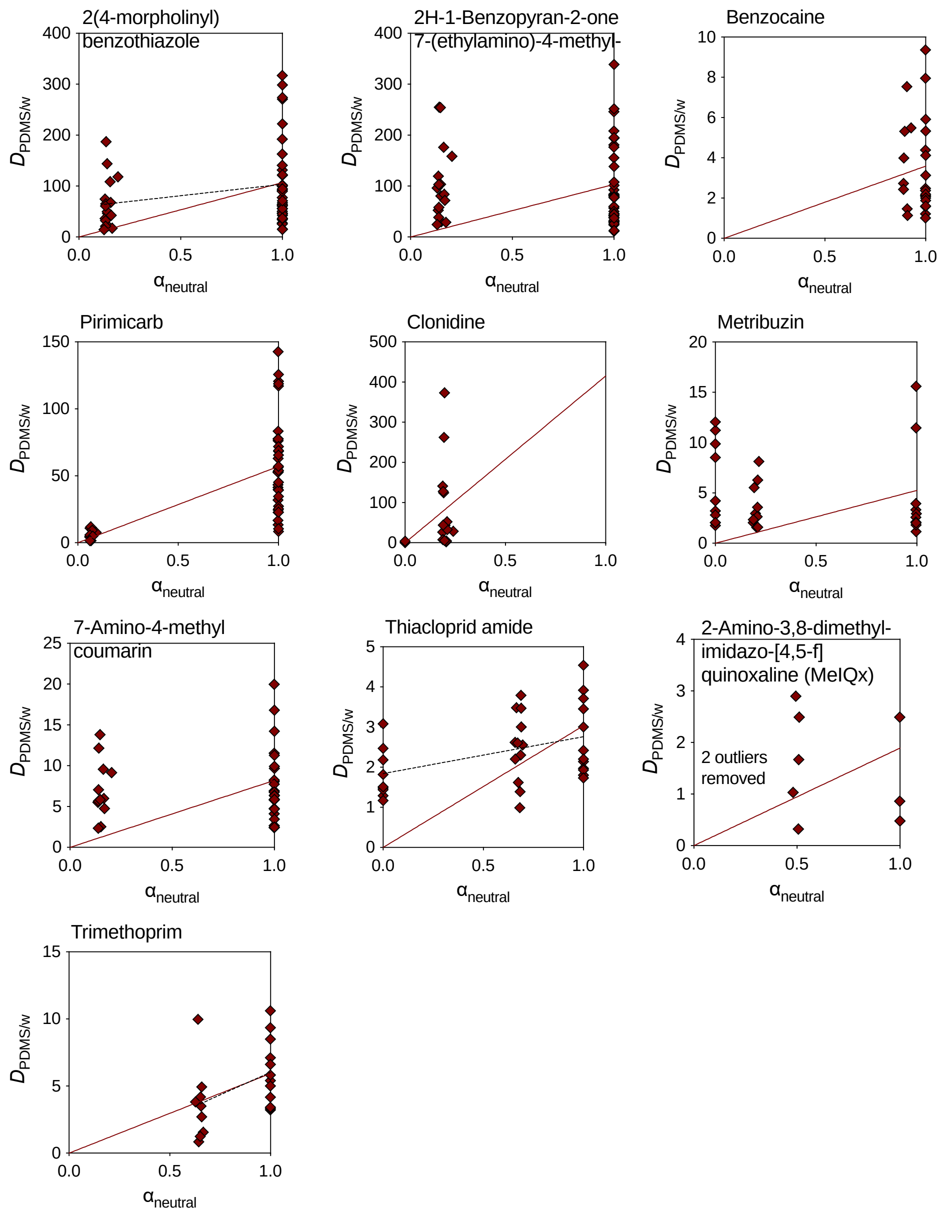

Figure S8 continued. The measured polydimethylsiloxane-water distribution ratios ($D_{PDMS/w}$) of monoprotic bases as a function of their neutral fractions ($\alpha_{neutral}$) at different pH levels. The red line is the best fit with an intercept of 0, the broken black line is the linear regression with the intercept as fit parameter (not in all cases possible). The based are sorted from high hydrophobicity to low hydrophobicity (expressed as $\log K_{ow}$ of the neutral species).

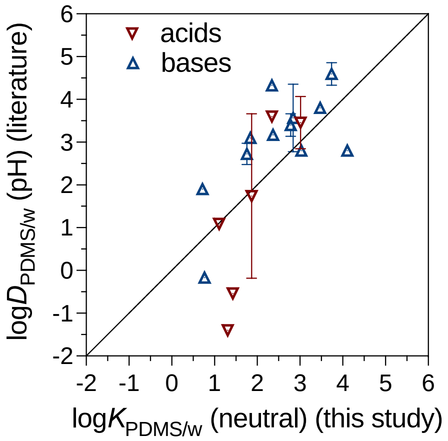

Figure S9. Comparison of mean of the polydimethylsiloxane-water partition constant ($K_{PDMS/w}$) of the neutral species of acids (down-facing red triangles) and bases (up-facing blude triangles) with published literature data ($D_{PDMS/w}$(pH)) that often did not specific the pH or was between pH 6 and 7.8.[1, 3-10, 12, 13] All data are reported in Table S5.

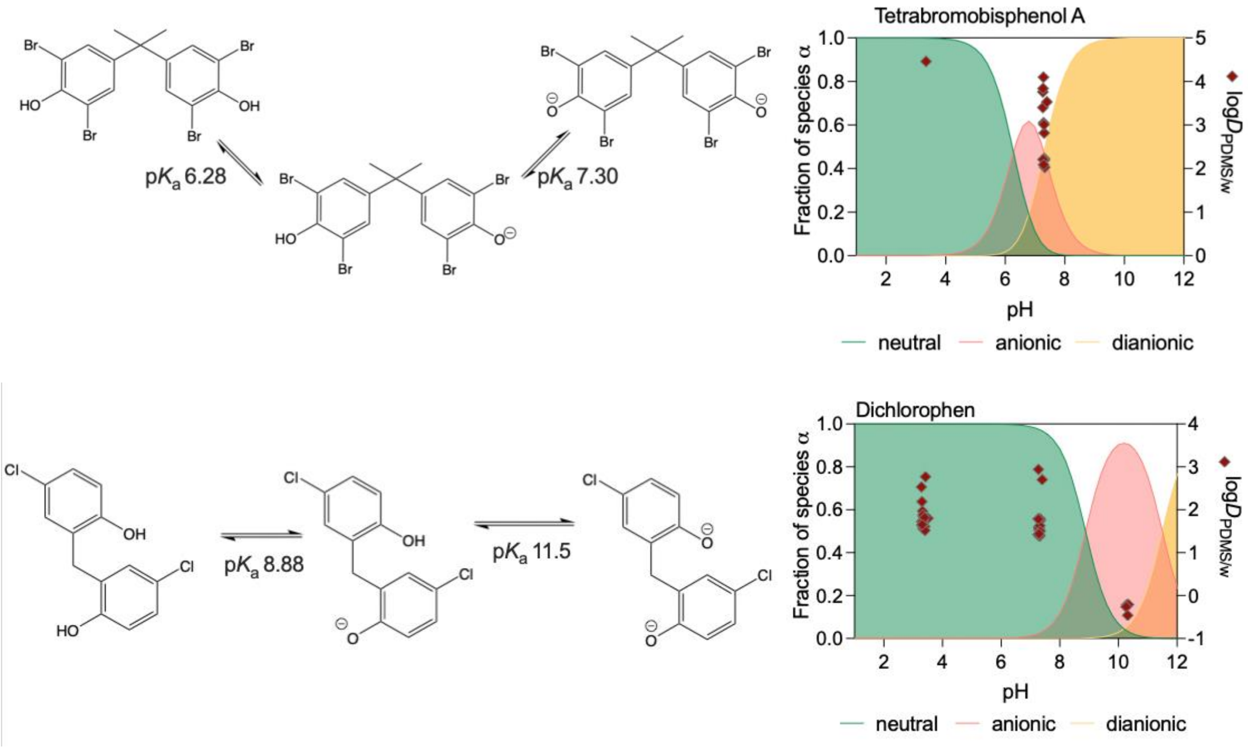

Figure S10. The fractions of all relevant species α_i of the diprotic acids as a function of pH (left y-axis, colored areas) and the measured polydimethylsiloxane-water distribution ratios (log$D_{PDMS/w}$, Table S4) (right y-axis, diamond symbols). The pK_a values were measured with the UV-metric method using the Sirius T3 (Table S7). The fractions α of the three species were calculated with eq. 8-10.

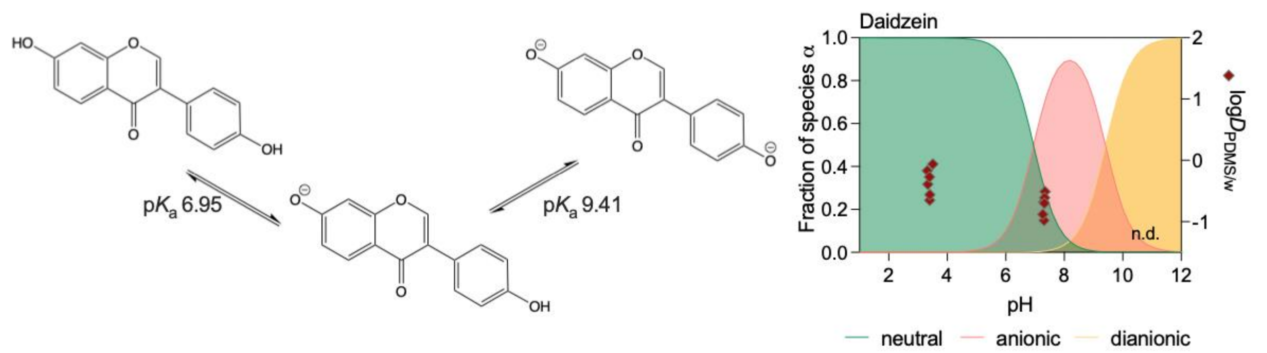

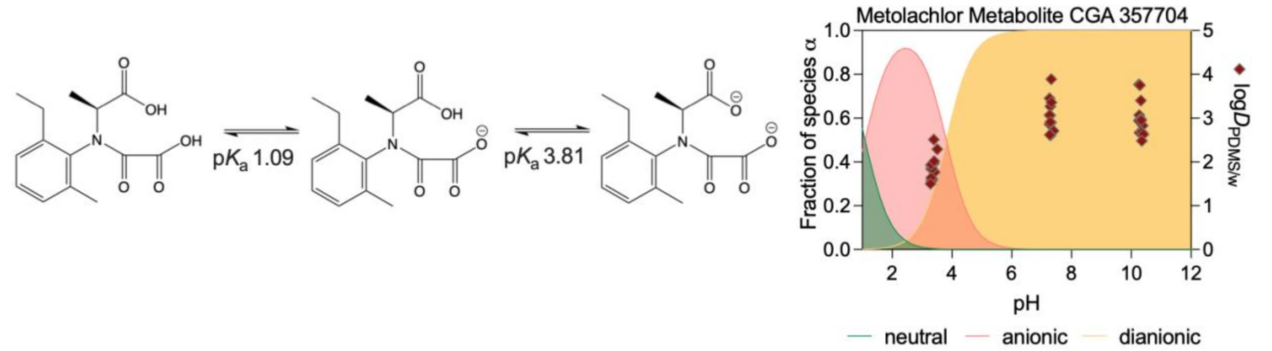

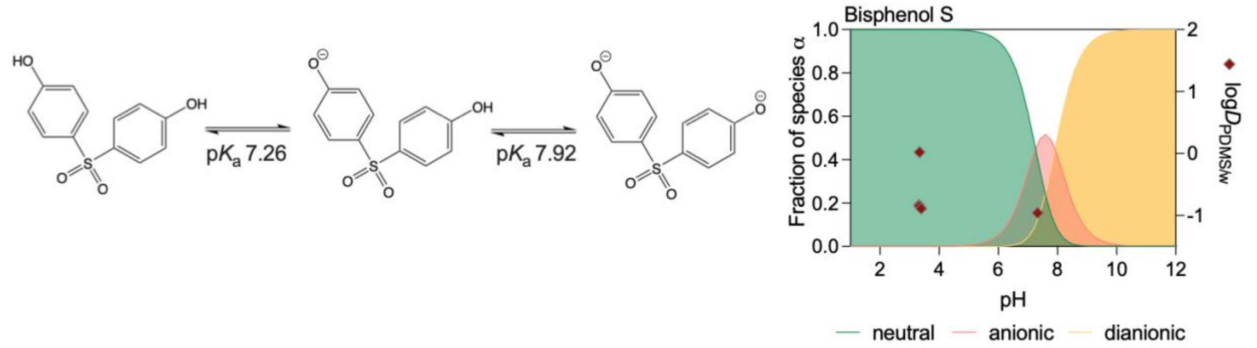

Figure S10 continued. The fractions of all relevant species α_i of the diprotic acids as a function of pH (left y-axis, colored areas) and the measured polydimethylsiloxane-water distribution ratios (log$D_{PDMS/w}$, Table S4) (right y-axis, diamond symbols). The pK_a values were measured with the UV-metric method using the Sirius T3 (Table S7). The fractions α_i of the three species were calculated with eq. 8-10.

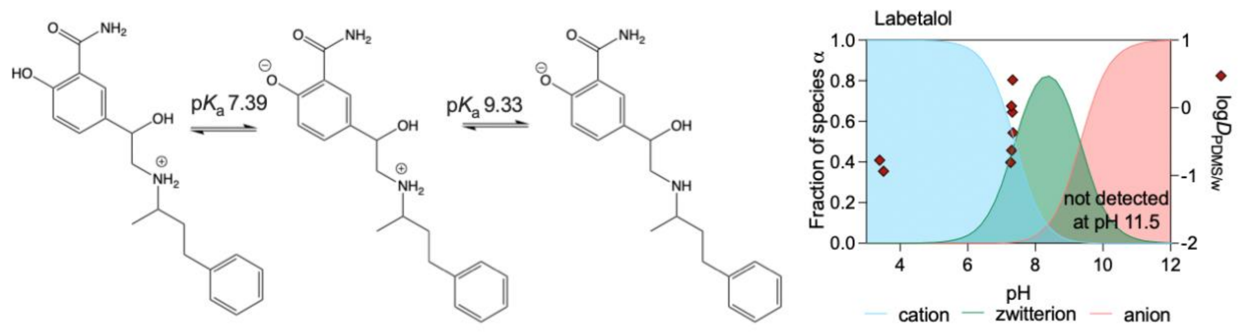

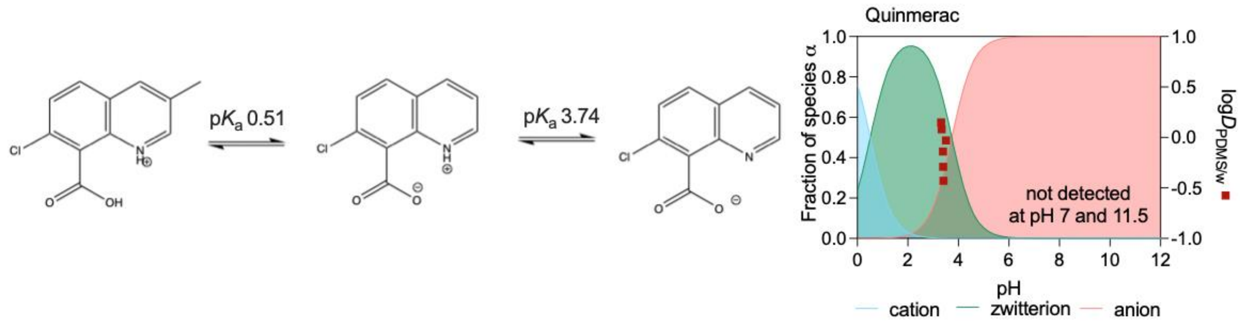

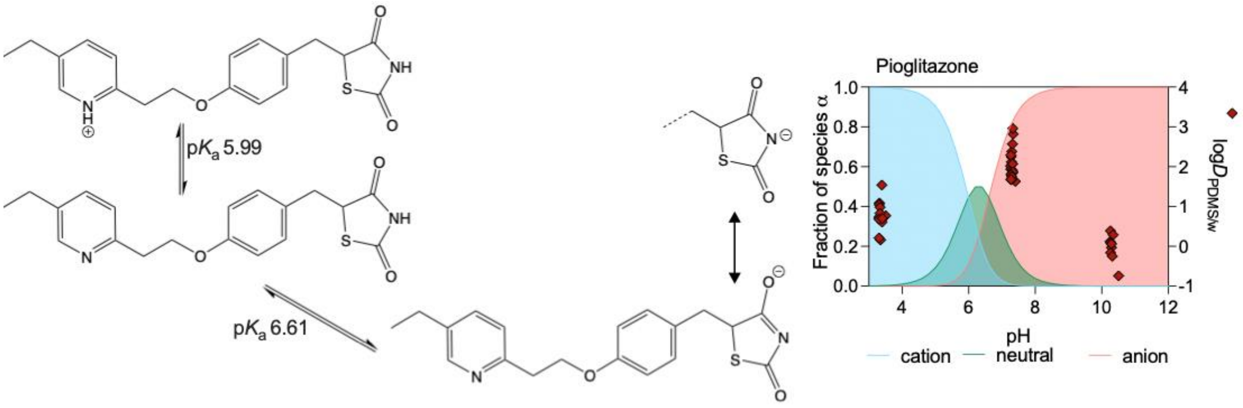

Figure S11. The fractions of all relevant species α_i of diprotic acid/bases as a function of pH (left y-axis, colored areas) and the measured polydimethylsiloxane-water distribution ratios (log$D_{PDMS/w}$, Table S4) (right y-axis, diamond symbols). The pK_a values were measured with the UV-metric method using the Sirius T3 (Table S7). The fractions α_i of the three species were calculated with eq. 8-10.

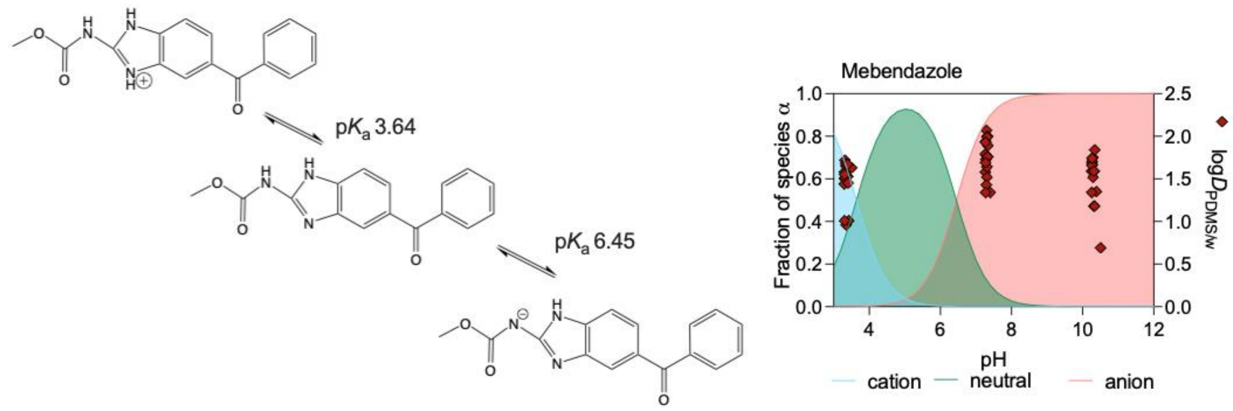

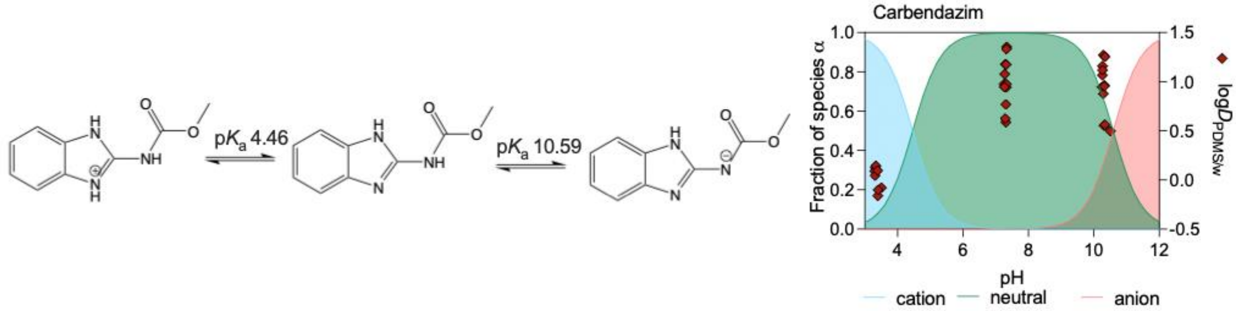

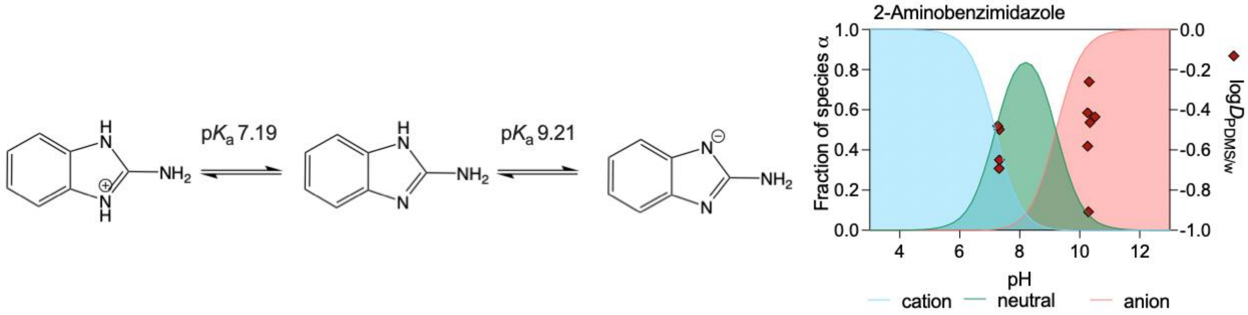

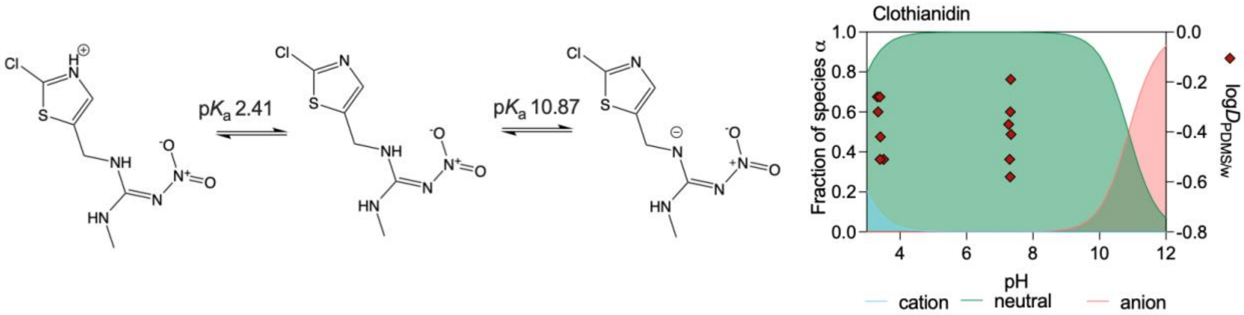

Figure S11 continued. The fractions of all relevant species α_i of diprotic acid/bases as a function of pH (left y-axis, colored areas) and the measured polydimethylsiloxane-water distribution ratios (log$D_{PDMS/w}$, Table S4) (right y-axis, diamond symbols). The pK_a values were measured with the UV-metric method using the Sirius T3 (Table S7). The fractions α_i of the three species were calculated with eq. 8-10.

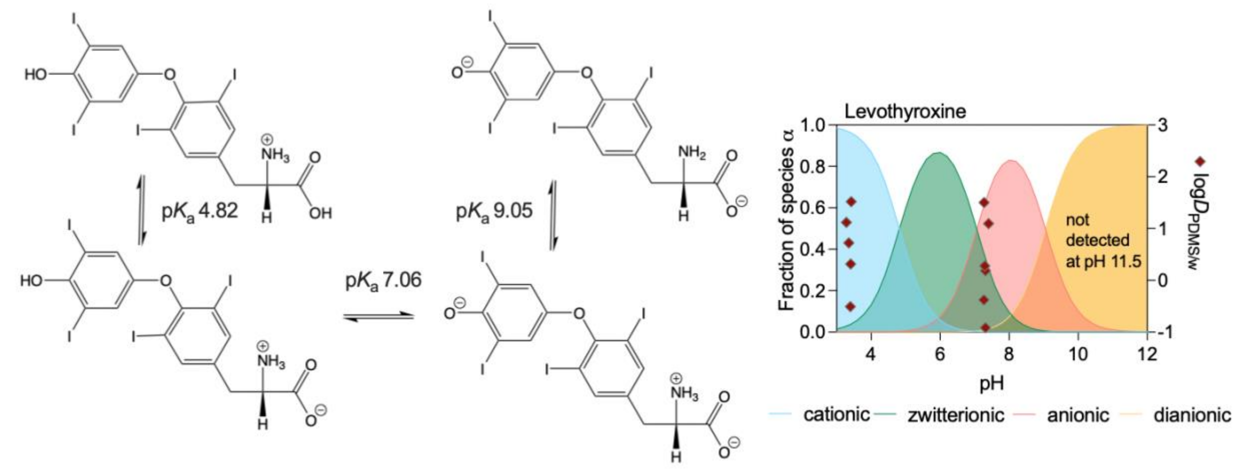

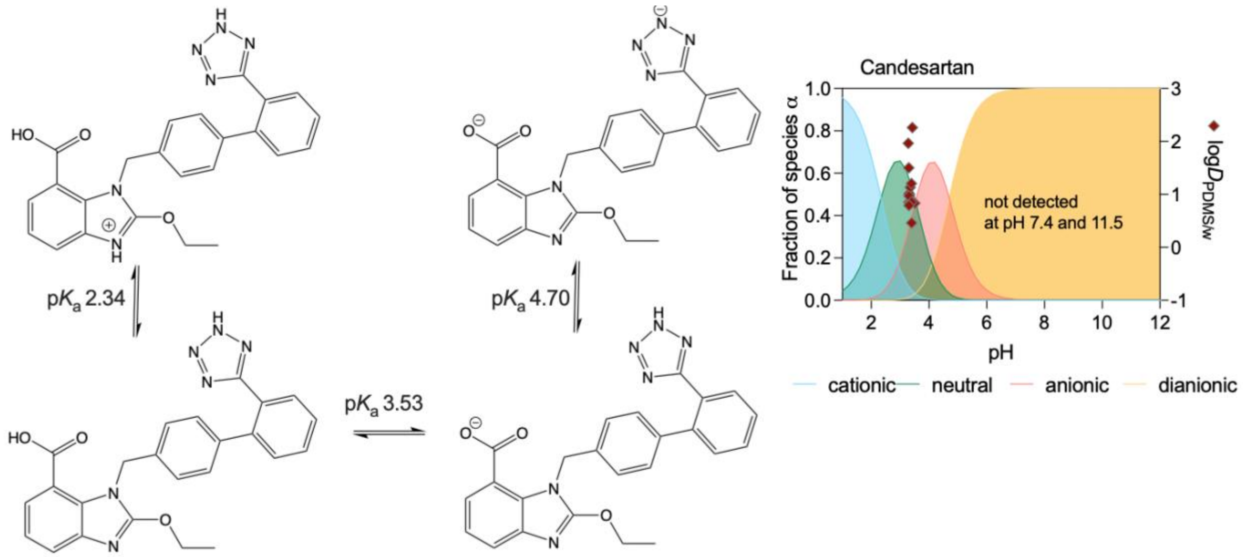

Figure S12. The fractions of all relevant species α_i of levothyroxine, two triprotic IOC (ionizable organic chemical) with two acidic and one basic functional group as a function of pH (left y-axis, colored areas) and the measured polydimethylsiloxane-water distribution ratios (log$D_{PDMS/w}$, Table S4) (right y-axis, diamond symbols). The pK_a values were measured with the UV-metric method using the Sirius T3 (Table S7). The fractions α_i of the four species were calculated with the equations given by Escher et al.[14]

References

1. Ahrens, L.; Daneshvar, A.; Lau, A. E.; Kreuger, J., Characterization of five passive sampling devices for monitoring of pesticides in water. *J Chromatogr A* **2015,** *1405*, 1-11.
2. Kwon, J. H.; Wuethrich, T.; Mayer, P.; Escher, B. I., Dynamic permeation method to determine partition coefficients of highly hydrophobic chemicals between poly(dimethylsiloxane) and water. *Anal. Chem.* **2007,** *79*, 6816-6822.
3. Magnér, J. A.; Alsberg, T. E.; Broman, D., Evaluation of poly(ethylene-co-vinyl acetate-co-carbon monoxide) and polydimethylsiloxane for equilibrium sampling of polar organic contaminants in water. *Environ. Toxicol. Chem.* **2009,** *28*, 1874-1880.
4. Martin, A.; Margoum, C.; Randon, J.; Coquery, M., Silicone rubber selection for passive sampling of pesticides in water. *Talanta* **2016,** *160*, 306-313.
5. Martin, A.; Margoum, C.; Jolivet, A.; Assoumani, A.; El Moujahid, B.; Randon, J.; Coquery, M., Calibration of silicone rubber rods as passive samplers for pesticides at two different flow velocities: Modeling of sampling rates under water boundary layer and polymer control. *Environ Toxicol Chem* **2018,** *37*, 1208-1218.
6. Neale, P. A.; Antony, A.; Gernjak, W.; Leslie, G.; Escher, B. I., Natural versus wastewater derived dissolved organic carbon: Implications for the environmental fate of organic micropollutants. *Water Res.* **2011,** *45*, 4227-4237.
7. Paschke, A.; Brummer, J.; Schuurmann, G., Silicone rod extraction of pharmaceuticals from water. *Anal. Bioanal. Chem.* **2007,** *387*, 1417-1421.
8. Pintado-Herrera, M. G.; Lara-Martín, P. A.; González-Mazo, E.; Allan, I. J., Determination of silicone rubber and low-density polyethylene diffusion and polymer/water partition coefficients for emerging contaminants. *Environ. Toxicol. Chem.* **2016,** *35*, 2162-2172.
9. Smedes, F., Silicone-water partition coefficients determined by cosolvent method for chlorinated pesticides, musks, organo phosphates, phthalates and more. *Chemosphere* **2018,** *210*, 662-671.
10. Sprunger, L.; Proctor, A.; Acree, W. E., Jr.; Abraham, M. H., Characterization of the sorption of gaseous and organic solutes onto polydimethyl siloxane solid-phase microextraction surfaces using the abraham model. *J. Chromatogr. A* **2007,** *1175*, 162-173.
11. Verhagen, R.; O'Malley, E.; Smedes, F.; Mueller, J. F.; Kaserzon, S., Calibration parameters for the passive sampling of organic uv filters by silicone; diffusion coefficients and silicone-water partition coefficients. *Chemosphere* **2019,** *223*, 731-737.
12. Wille, K.; Claessens, M.; Rappe, K.; Monteyne, E.; Janssen, C. R.; De Brabander, H. F.; Vanhaecke, L., Rapid quantification of pharmaceuticals and pesticides in passive samplers using ultra high performance liquid chromatography coupled to high resolution mass spectrometry. *J. Chromatogr. A* **2011,** *1218*, 9162-9173.
13. Zambonin, C. G.; Palmisano, F., Determination of triazines in soil leachates by solid-phase microextraction coupled to gas chromatography-mass spectrometry. *J. Chromatogr. A* **2000,** *874*, 247-255.
14. Escher, B.; Abagyan, R.; M, E.; Klüver, N.; Redman, A.; Zarfl, C.; Parkerton, T., Recommendations for improving methods and models for aquatic hazard assessment of ionizable organic chemicals. *Environ. Toxicol. Chem.* **2020,** *39*, 269-286.
